# Surface Processes Control the Fate of Reactive Oxidants Generated by Electrochemical Activation of Hydrogen Peroxide on Stainless-Steel Electrodes

**DOI:** 10.1021/acs.est.2c08404

**Published:** 2023-03-16

**Authors:** Yanghua Duan, Wenli Jiang, David L. Sedlak

**Affiliations:** Department of Civil & Environmental Engineering University of California, Berkeley, Berkeley, California 94720, United States

**Keywords:** advanced oxidation process, one-electron reduction, electrification, three-dimensional electrode, electro-Fenton

## Abstract

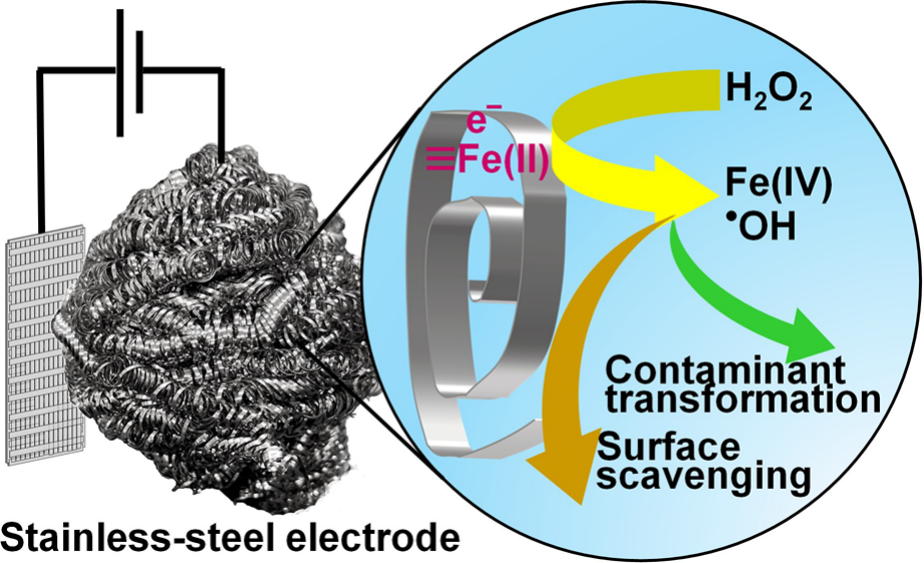

Low-cost stainless-steel electrodes can activate hydrogen peroxide (H_2_O_2_) by converting it into a hydroxyl radical (^•^OH) and other reactive oxidants. At an applied potential of +0.020 V, the stainless-steel electrode produced ^•^OH with a yield that was over an order of magnitude higher than that reported for other systems that employ iron oxides as catalysts under circumneutral pH conditions. Decreasing the applied potential at pH 8 and 9 enhanced the rate of H_2_O_2_ loss by shifting the process to a reaction mechanism that resulted in the formation of an Fe(IV) species. Significant metal leaching was only observed under acidic pH conditions (i.e., at pH <6), with the release of dissolved Fe and Cr occurring as the thickness of the passivation layer decreased. Despite the relatively high yield of ^•^OH production under circumneutral pH conditions, most of the oxidants were scavenged by the electrode surface when contaminant concentrations comparable to those expected in drinking water sources were tested. The stainless-steel electrode efficiently removed trace organic contaminants from an authentic surface water sample without contaminating the water with Fe and Cr. With further development, stainless-steel electrodes could provide a cost-effective alternative to other H_2_O_2_ activation processes, such as those by ultraviolet light.

## Abstract 2

The efficiency of transformation of organic contaminants by H_2_O_2_ on stainless-steel electrodes is strongly affected by surface scavenging of oxidants.

## Introduction

To enable the use of advanced oxidation processes in small-scale systems (e.g., point-of-use drinking water treatment), treatment units must be capable of converting soluble oxidants (e.g., H_2_O_2_, O_3_, HOCl) into species that react with recalcitrant compounds (e.g., ^•^OH, ^•^Cl).^1,2^ Ultraviolet light is one of the most popular methods of oxidant activation in water recycling, groundwater remediation, and industrial wastewater treatment because it produces few toxic byproducts and does not introduce ions that could diminish water quality.^3,4^ However, activation by ultraviolet light is often limited by competition for photons with other dissolved species. For small-scale systems, deposition of minerals on the surfaces of submerged lamps or loss of light emitted by suspended lamps through surface reflection further complicates the activation process.^5^ As an alternative, the activation of H_2_O_2_ by Fe(II) released from the oxidation of an iron anode has been used for industrial wastewater treatment.^6^ Such electro-Fenton systems are impractical for drinking water and many other applications because they only produce high yields of ^•^OH over a relatively narrow acidic pH range (i.e., pH 2–4) due to a shift in the reaction mechanism from one-electron processes that produce ^•^OH to more selective oxidants (e.g., Fe[IV]) as the pH increases.^7−10^ Use of electro-Fenton systems is also complicated by the need to collect and dispose of the Fe(III)-containing waste produced during the activation process.^6^

To overcome these challenges, researchers have attempted to develop heterogeneous catalysts capable of efficiently converting H_2_O_2_ to ^•^OH and other strong oxidants under circumneutral pH conditions.^11,12^ Despite considerable progress in increasing the rate of H_2_O_2_ activation, most of the oxidants produced by the catalytic activation lack the high reactivity and low selectivity of ^•^OH (i.e., <5% of the transformed H_2_O_2_ produces species capable of oxidizing compounds that are typically used as ^•^OH probes)^13−20^ presumably because nonradical activation mechanisms convert H_2_O_2_ directly into O_2_ and H_2_O through two-electron transfer reactions or produce weaker, more selective oxidants, like Fe(IV).^21−24^ Although heterogeneous Fenton catalysts containing various additives (e.g., Si and Al) sometimes exhibit higher apparent ^•^OH yields,^25^ their applications are still limited by instability of the catalysts.^26^ Custom-made electrodes composed of carbonaceous materials and metals^27−31^ have been proposed as alternatives to heterogeneous catalysts, but their fabrication requires the use of flat plate reactors that exhibit slow kinetics due to poor mass transfer properties.^32−34^

Recently, Weng et al. (2020) reported that an inexpensive stainless-steel mesh material converts H_2_O_2_ into oxidants capable of transforming 1,4-dioxane.^35^ This material is particularly attractive for use in modular electrochemical systems because it has the potential to function as a three-dimensional electrode, which could enhance the transport of oxidants and contaminants to the electrode surface.^36^ However, the initial study only considered extremely pure water (i.e., reverse osmosis permeate) maintained at pH 5.6 with 2 mM phosphate buffer.

To assess the potential for using stainless-steel electrodes to transform organic contaminants by activating H_2_O_2_ under conditions likely to be encountered in small-scale water treatment systems, we evaluated the performance of a three-dimensional stainless-steel electrode under environmentally relevant conditions. By evaluating the effects of pH and potential on rates of H_2_O_2_ loss and the ability of oxidants produced from H_2_O_2_ reduction to oxidize different probe compounds, we were able to optimize the activation conditions. We also assessed the role of contaminant concentration on oxidant utilization efficiency in the presence of natural organic matter and other species that are typically responsible for the loss of reactive species in advanced oxidation processes.

## Materials and Methods

### Materials

All experiments were performed at room temperature (23 ± 2 °C) with chemicals of reagent grade or higher (Sigma-Aldrich, St. Louis, MO, and Fisher Scientific, Pittsburgh, PA). Ultrapure water from a Milli-Q system (*R* > 18 MΩ) was used for all experiments.

### Electrolysis

Electrolysis experiments were performed at fixed potentials versus a Ag/AgCl reference electrode (3 M NaCl, BASi, West Lafayette, IN) controlled by a multichannel potentiostat (Gamry Instruments Inc., Warminster, PA). All potentials are reported versus a standard hydrogen electrode (SHE). Electrolysis experiments were performed in the batch mode in a glass H-cell reactor (Figure S1, *V*_cathode_ = *V*_anode_ = 100 mL) that was separated by an anion-exchange membrane (Fumasep FAS-PET-130, The Fuel Cell Store, College Station, TX). The anion-exchange membrane minimized the transport of H_2_O_2_ and metal ions between the two chambers. A preconditioned stainless-steel electrode was used as the working electrode (Text S1) and a platinum electrode was used as the counter electrode (1.0 × 1.0 cm, 99.99% Pt).

The working electrode chamber was filled with 200 mM Na_2_SO_4_ amended with 1 mM buffer and different concentrations of probe compounds (e.g., methanol or 2-propanol). The addition of an inert electrolyte (e.g., Na_2_SO_4_) avoided internal resistance compensation and a stabilization step,^35,37^ but would not be needed if the system were to be scaled up to a working prototype with a small interelectrode distance and without an ion-exchange membrane. The following buffers (1 mM) were used: sodium acetate (pH 4 and 5), 2-(N-morpholino)ethanesulfonic acid (MES; pH 6), piperazine-N,N′-bis(ethanesulfonic acid) (PIPES; pH 7), and sodium borate (pH 8 and 9). MES, PIPES and borate do not form complexes with Fe(II) or Fe(III).^38^ Under most conditions, the pH values varied by less than 0.2 pH units, with the greatest changes observed under acidic conditions (e.g., pH 4 and 5) in which the solution pH changed by about 0.3 pH units.

Methanol was employed as the probe compound for oxidants because it is uncharged over the pH range of interest, and the main product of methanol oxidation by both ^•^OH and Fe(IV), formaldehyde, can be quantified easily.^7^ 2-Propanol was chosen as the other oxidant probe because it is only oxidized to acetone by ^•^OH and other strong oxidants and is not expected to interact with the electrode surface.^7^ Because ^**•**^OH is expected to be produced at the electrode surface and the relative reactivity of the surface-bound ^•^OH with organic compounds as well as the extent to which the radical diffuses into solution are unknown,^39^ the reactive oxidant that can oxidize both methanol and 2-propanol was referred to as ^•^OH, without differentiating between ^•^OH adsorbed on the electrode surface or ^•^OH reacting in the solution. Control experiments showed that H_2_O_2_ did not react with methanol or 2-propanol over the pH range tested in this study (Figure S3).

To assess the overall yield of oxidants from H_2_O_2_ activation, a high concentration of probe compounds (i.e., 100 mM) was used. Under these conditions, the yield of reactive oxidants generated could be quantified from the formation of the oxidation products (i.e., formaldehyde and acetone):
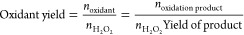
1where the yield of product (i.e., formaldehyde and acetone) for the reactions between the probe compounds and ^**•**^OH are 1.000 for methanol and 0.867 for 2-propanol.^40^ The concentrations of probe compounds in the electrolyte did not affect H_2_O_2_ activation rates or observed current densities (Figure S4).

Methanol was chosen as a surrogate for ^•^OH-scavenging compounds to assess the scavenging of ^•^OH by the electrode surface using a competition kinetics method because of the ease of quantification of its oxidation product relative to that of 2-propanol. Because methanol does not interact with surfaces to an appreciable extent,^7^ the reaction rate constant with aqueous ^•^OH (*k*_CH_3_OH,•OH_ = 9.7 × 10^8^ M^–1^ s^–1^) was used to estimate the rate constant for reaction between the electrode surface and ^•^OH.^41^

Electrolysis experiments were also conducted to assess the transformation of trace organic contaminants under conditions relevant to water treatment (Text S2). In these experiments, an undivided reactor was used to minimize pH changes during electrolysis (Figure S5). Carbamazepine and atrazine (20 μg/L) were used as representative trace organic contaminants because of their high frequencies of detection in surface water and their relative resistance to oxidants other than ^•^OH.^42−44^

### Analytical Methods

Samples (6 mL aliquots) were periodically collected from the stainless-steel electrode chamber for the analysis of H_2_O_2_, metals (i.e., dissolved Fe and Cr), and oxidation products. Details of the analytical methods are listed in Text S3.

## Results and Discussion

### Effects of pH and Applied Potential on H_2_O_2_ Transformation Rates

**Figure 1:**
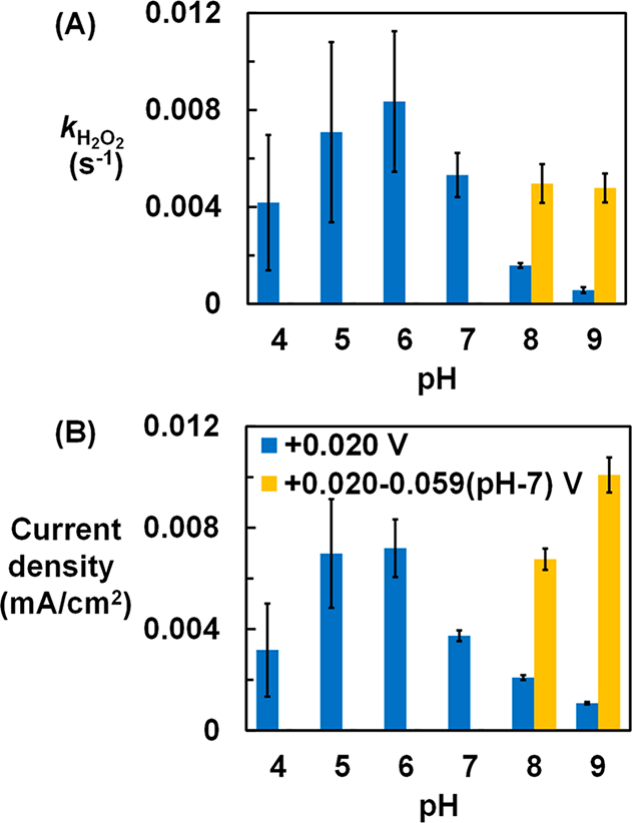
(A) Observed first-order rate constants for H_2_O_2_ decomposition, and (B) observed current densities at different pH values and applied potentials. [H_2_O_2_]_0_ = 1.25 mg/L, [Probe compound] = 100 mM. Error bars represent one standard deviation. Surface area of the stainless-steel electrode was 360 cm^2^.

Experiments were conducted in the pH range 4–9 with an initial H_2_O_2_ concentration of 1.25 mg/L (i.e., 37 μM) and an applied potential of +0.020 V. These values had been identified previously as the optimal initial H_2_O_2_ concentration and potential for organic contaminants (i.e., 1,4-dioxane) removal in experiments conducted in phosphate-buffered solutions (pH 5.8).^35^ H_2_O_2_ decomposition rates doubled as the pH increased from 4 to 6. As the pH values increased from 6 to 9, the H_2_O_2_ decomposition rates decreased by over an order of magnitude (Figure 1A, Figure S8). Current densities (Figure 1B) followed a similar trend for H_2_O_2_ decomposition, with the highest current density observed at pH 6. The large uncertainties observed for both H_2_O_2_ decomposition rates and current densities at pH values below 7 were likely associated with changes in the electrode surface that took place during the experiment (details are described in a later section).

The applied potentials in all experiments were above the H_2_ evolution potential (Figure S9), indicating that the reduction of water was not responsible for the observed trends. According to the Nernst equation, the equilibrium potential of the one-electron reduction of H_2_O_2_ to ^**•**^OH (eq 2) varied with pH, with a slope of −0.059 V per unit increase in pH. Thus, the overpotential driving this reaction (i.e., difference between the operating potential and the equilibrium potential) decreased as the pH increased. Therefore, the slow reaction kinetics at pH 8 and 9 could have been due to the smaller driving forces (i.e., overpotentials) for H_2_O_2_ activation.
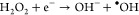
2To further investigate the effect of overpotential on the performance of the stainless-steel electrode at high pH values (i.e., 8 and 9), applied potentials were adjusted by −0.059 V per pH increment (i.e., −0.039 V at pH 8 and −0.098 V at pH 9) to maintain the same driving force as values applied at pH 7 and +0.020 V. With the same overpotential, H_2_O_2_ decomposition rates were almost identical to those observed at pH 7 (yellow bars in Figure 1). However, the observed current densities were higher than those observed at pH 7 and +0.020 V, suggesting that H_2_O_2_ could have been lost through another pathway as the pH increased.

To gain insight into the rate-limiting step of the H_2_O_2_ activation process, the experiment was repeated at varying initial H_2_O_2_ concentrations. In comparison with the experiments conducted with 1.25 mg/L H_2_O_2_, increasing the initial H_2_O_2_ concentration to 2.5 and 5.0 mg/L resulted in about a 10% and a 40% reduction in the H_2_O_2_ loss rates (Figure S10). The decrease in the rate of H_2_O_2_ decomposition was much smaller than values expected if H_2_O_2_ activation on the surface was the rate-limiting step (i.e., 50% and 75% reduction estimated based on the basis of zero-order kinetics). Therefore, the decrease in the H_2_O_2_ decomposition rate was likely caused by factors other than saturation of the reactive surface sites at an elevated H_2_O_2_ concentration. Rather, the measured rate constants were consistent with mass-transport limitations (predicted *k*_limt_ = 5.4 × 10^–3^ s^–1^, Text S4). Finally, a linear relationship was observed between current densities and initial H_2_O_2_ concentrations, suggesting the presence of excess available reactive sites on the stainless-steel electrode surface (Figure S11). From these results, we conclude that the transport of H_2_O_2_ to the electrode surface was likely the rate-limiting step for H_2_O_2_ activation.

### Reactive Oxidant Production

Although the activation of H_2_O_2_ by Fe(II) and Fe oxides has been studied for more than a century,^45^ the identity of the reactive oxidants produced in these systems is still unresolved. Most evidence suggests that the homogeneous reaction of uncomplexed Fe(II) and H_2_O_2_ produces ^•^OH at low pH values and that a different oxidant, most likely an Fe(IV) species, is produced at pH values above 4.^8,46^ Fe(IV) species also appear to dominate in the presence of ligands such as EDTA^47^ and when Fe(II) is produced by the photolysis of Fe(III) complexes (i.e., the photo-Fenton reaction).^48^ For heterogeneous processes, the widely accepted mechanism involves ^•^OH production through redox reactions between surface iron species (i.e., ≡Fe[II]).^49^ However, the identity of the reactive species generated with other heterogeneous catalysts is less certain. For example, both ^•^OH and an unidentified weak oxidant were formed over the acidic to near-neutral pH in a ferrihydrite-induced heterogeneous Fenton process.^24^

**Figure 2:**
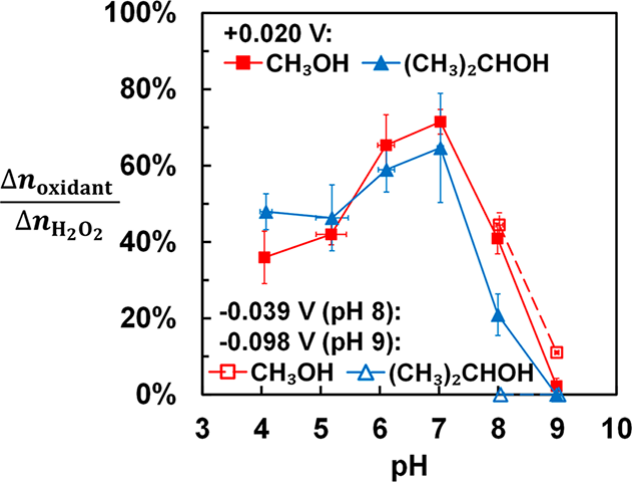
Yield of oxidants as a function of pH and applied potential. [H_2_O_2_]_0_ = 1.25 mg/L, [Probe compound] = 100 mM. Error bars represent one standard deviation; error bars not shown are smaller than symbols.

At + 0.020 V, the yield of the oxidation products (i.e., formaldehyde or acetone) increased as the pH increased from 4 to 7 and decreased as the pH further increased from 7 to 9 (Figure 2). The oxidant species generated at +0.020 V transformed similar amounts of methanol and 2-propanol at pH values of 7 and below, indicating that ^•^OH was the main oxidant species. A maximum yield for ^•^OH production of 71% was observed at pH 7, which is over an order of magnitude higher than the yields observed for heterogeneous Fenton systems.^13−20^ At pH 8, the amount of 2-propanol transformed by the oxidants was about half that of methanol, indicating that H_2_O_2_ was converted into both ^•^OH and Fe(IV) in approximately equal amounts. At pH 9, nearly no reactive oxidants were detected when +0.020 V was applied despite a loss of about 20% of the H_2_O_2_ during the experiments.

After the applied potential was lowered, the total yield of reactive oxidants observed at pH 8 was about the same as those observed at +0.020 V and the total yield of reactive oxidants at pH 9 increased from 2% to 11% (hollow red symbols versus solid red symbols in Figure 2). However, the reactive oxidants produced under these conditions were capable of oxidizing only methanol (hollow red symbols versus hollow blue symbols in Figure 2), indicating Fe(IV) was being produced.

The electron utilization efficiencies for producing reactive oxidants (Figure S12A) and for activating H_2_O_2_ (Figure S12B) were similar to those of the yield of oxidants, with the maximum values observed at pH 7. At the optimal operating conditions (i.e., pH 7 and +0.020 V), about 50% of electrons were consumed for H_2_O_2_ production. Most of the remaining electrons were accounted for by O_2_ reduction, as determined by results from a control experiment conducted in a deaerated electrolyte solution (Figure S11).

### Metal Release from Stainless-Steel Electrodes

**Figure 3:**
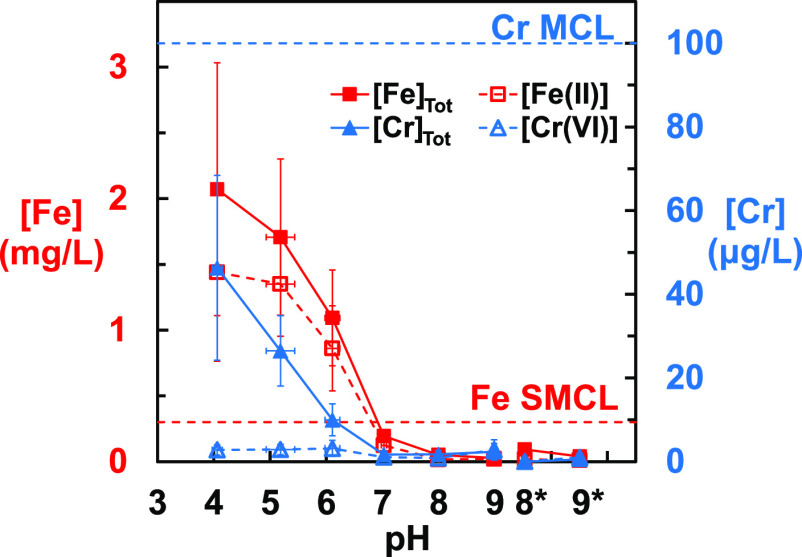
Concentrations of Fe and Cr after 5 min of electrolysis at a potential of +0.020 V. The * symbol represents experiments conducted at potentials lower than +0.020 V (i.e., −0.039 V at pH 8 and −0.098 V at pH 9). Error bars represent one standard deviation.

The leaching of Fe and Cr from preconditioned stainless-steel electrodes decreased as the pH value increased from pH 4 to 6 at +0.020 V. At pH 7 and above, metal concentrations decreased by over an order of magnitude relative to values observed under acidic conditions (Figure 3 and Figure S13). Fe(II) accounted for approximately 80% of the Fe detected in solution, and Cr(III) accounted for over 90% of the chromium released from the electrodes. The concentration of Cr(VI) was near or below the limit of detection (i.e., 2 μg/L) under all conditions. Although negatively charged Cr(VI) species (e.g., HCrO_4_^–^) can cross the anion exchange membranes, Cr was not detected in the anode chamber. The chromium concentrations observed during these experiments were always below the USEPA maximum contaminant level (MCL) for total Cr of 100 μg/L^50^ and the California MCL for chromium(VI) of 10 μg/L.^51^ However, the Fe concentrations were above the secondary maximum contaminant level (SMCL) of 0.3 mg/L^52^ in experiments conducted at pH 6 and below. Other metals present in 410 stainless-steel (i.e., Ni and Mg) were not detected in solution under any pH conditions.

Under conditions that the stainless-steel electrode is most likely to be deployed for water treatment (e.g., pH 6 and 7), a total of about 25 μM ^•^OH was produced as a result of the release of metals depicted in Figure 3. The produced ^•^OH had been proven to be sufficient to achieve 0.5 log removal of 1,4-dioxane, a benchmark for advanced oxidation processes, from reverse osmosis permeate.^35^

Some aspects of the leaching of metals can be understood by considering the thermodynamics of the passivation layer (Figure S9). At pH 4 and 5 and an electrode potential of +0.020 V, the Fe(III)-oxides in the passivation layer should be reduced to Fe(II) (the system was undersaturated with respect to Fe(OH)_2(S)_ based on Fe(II) concentrations measured in the aqueous phase), and the Cr(III)-oxides in the passivation layer should have been released as aqueous Cr(III) species (i.e., Cr^3+^ or Cr(OH)^2+^). Under these acidic conditions, the passivation layer should have dissolved, exposing elemental Fe and Cr to the solution. The exposed elemental Fe and Cr are prone to oxidation and should also have resulted in the formation of Fe(II) and Cr(III) species, which was consistent with the modest anodic current observed at +0.020 V during the preconditioning process (Figure S2F). At pH 6 and an electrode potential of +0.020 V, a passivation layer consisting of Fe(III)-oxides and Cr(III)-oxides could have been reduced to form FeCr_2_O_4_ (eq 5 in Table S2). The transformation to a new mineral species might explain the release of dissolved Fe and Cr observed at pH 6, especially because the newly formed amorphous FeCr_2_O_4_ would have a higher solubility than that of the crystalline form. At pH 7 and above, a stable passivation layer composed of Fe_2_O_3(S)_ and Cr_2_O_3(S)_ should have been formed on the surface of the stainless-steel electrode. This phase would prevent metal leaching from stainless-steel under circumneutral pH conditions.^53^

The oxidation state of Fe on the electrode surface was characterized by X-ray photoelectron spectroscopy with samples collected from at least three different locations on the electrode surface for each operating condition. More Fe(II) and Fe(0) was observed under the acidic pH conditions where metal leaching was observed (Figure S14), which was consistent with the reduction of Fe(III)-oxides on the passivation layer coating the electrode surface. The greater heterogeneity of the electrode surface observed under conditions in which metal leaching was observed (i.e., the wider standard deviations under acidic conditions) was consistent with the fact that corrosion likely takes place at different rates across the electrode surface.^54^

Increasing the initial concentrations of H_2_O_2_ (0–5 mg/L), methanol (5–100 mM), and dissolved O_2_ (deaerated versus air-saturated conditions) did not affect the release of Fe or Cr from stainless-steel electrodes (Figures S15 and S16). In fact, the presence of O_2_ and higher concentrations of H_2_O_2_ slightly lowered metal leaching, possibly by enhancing the rate of oxidation of surface ≡Fe(0) and ≡Fe(II) to form a more effective passivation layer. Therefore, H_2_O_2_ activation processes, reactions of the oxidant species with methanol, and O_2_ reduction were excluded as the cause of the observed metal leaching. At an open circuit potential, the stainless-steel did not leach any metals when in contact with H_2_O_2_ (Figure S15), further supporting the conclusion that metal leaching was caused by the electrochemically driven redox reactions on the electrode surface.

### Surface Scavenging of ^•^OH

**Figure 4:**
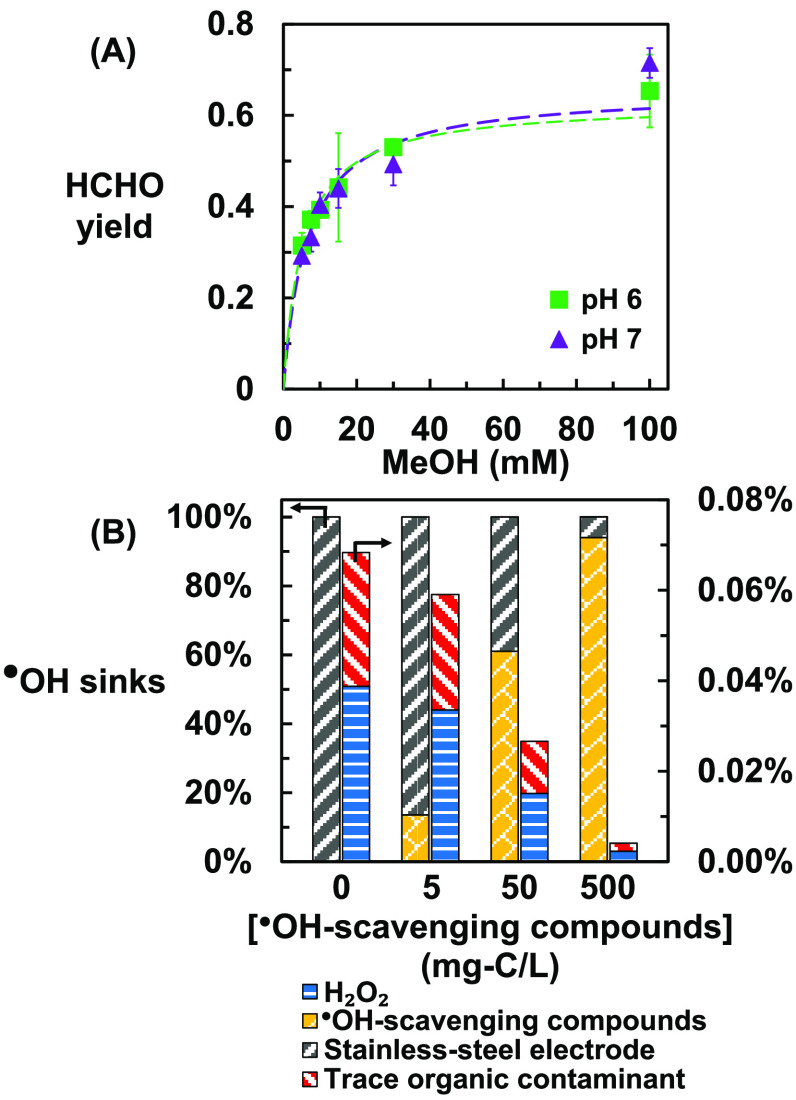
(A) Formaldehyde yield as a function of methanol concentration (*k*_CH_3_OH,•OH_ = 9.7 × 10^8^ M^–1^ s^–1^). Potential = +0.020 V; [H_2_O_2_]_0_ = 1.25 mg/L. Dashed lines represent the formaldehyde yield predicted based on the basis of eq 3. Error bars represent one standard deviation (*n* = 3–6); error bars not shown are smaller than symbols. (B) Predicted fate of ^•^OH for the treatment of 20 μg/L of a representative trace organic contaminant (*k*_•OH_ = 9 × 10^9^ M^–1^ s^–1^) with 4.5 g of stainless-steel electrode in the presence of 1.25 mg/L of H_2_O_2_ and various concentrations of ^•^OH-scavenging compounds (*k*_•OH_ = 8.1× 10^4^ L (mg of C)^−1^ s^–1^). The percentage of ^•^OH reacting with the electrode surface and ^•^OH-scavenging compounds (e.g., NOM) are indicated on the left axis, and reactions with representative trace organic contaminants and H_2_O_2_ are shown on the right axis.

Reactions taking place at the electrode surface can compete with organic contaminants for oxidants produced when H_2_O_2_ is activated, lowering the overall efficiency of the treatment process.^55,56^ To assess this effect, concentrations of methanol were varied from 5 to 100 mM (Figure 4A). The formaldehyde yield  was used to determine the fraction of oxidants produced upon H_2_O_2_ that could react with dissolved organic contaminants under different conditions. The formaldehyde yield depends on both the ^•^OH yield from the H_2_O_2_ activation process and the fraction of the ^•^OH that reacts with the probe compound (i.e., methanol).

Under the conditions employed in this study, ^•^OH produced at the electrode surface could react with methanol, other dissolved solutes (e.g., HCO_3_^–^, Fe^2+^, H_2_O_2_), or the electrode surface. If the concentration of methanol does not affect the rate of production of ^•^OH, the formaldehyde yield can be expressed as
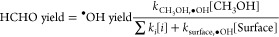
3where *i* represents the species in the aqueous phase (i.e., methanol, buffer, Fe^2+^, H_2_O_2_) that react with ^•^OH.

As indicated in Figure 4A, the yield of formaldehyde plateaued at about 70% (i.e., the maximum yield of ^•^OH produced on the stainless-steel surface, as depicted in Figure 2). At methanol concentrations above 30 mM, methanol outcompeted all other species for ^•^OH and the formaldehyde yield approached the maximum values. As methanol concentration decreased, the formaldehyde yield dropped, indicating significant competition from other ^•^OH scavenging species.

Results from these experiments were used to predict the fate of ^•^OH under conditions that might be encountered in treatment systems (i.e., see the caption for Figure 4B and Text S5 and S6 for details). Briefly, the reaction rate for ^•^OH scavenging by the electrode surface was assumed to be constant at all of the initial methanol concentrations and was calculated by using competition kinetics by assuming the methanol concentration did not affect the formation of ^•^OH (i.e., constant ^•^OH yield). The fraction of ^•^OH scavenged by the electrode surface increased from about 5% to 85% as the concentrations of ^•^OH-scavenging compounds decreased from 500 mg of C/L to 5 mg of C/L. These results suggest that the performance of the stainless-steel electrode should not be affected appreciably by the presence of competing organic compounds (i.e., dissolved organic carbon concentrations are typically <10 mg of C/L in water that would be treated in a point-of-use water treatment system). Furthermore, less than 0.1% of the ^•^OH produced by H_2_O_2_ activation is predicted to react with the trace organic contaminant under all conditions, irrespective of the concentrations of ^•^OH scavengers in the solution. This means that a relatively large amount of H_2_O_2_ is needed for contaminant transformation. Due to the modest role that other solutes play in determining the fraction of the ^•^OH that reacts with trace organic contaminants, the stainless-steel electrode may be nearly as effective in removing trace organic contaminants in industrial wastewater containing nontarget organic compounds that would scavenge ^•^OH in a conventional advanced oxidation process.

### Possible Mechanisms of H_2_O_2_ Activation

**Figure 5:**
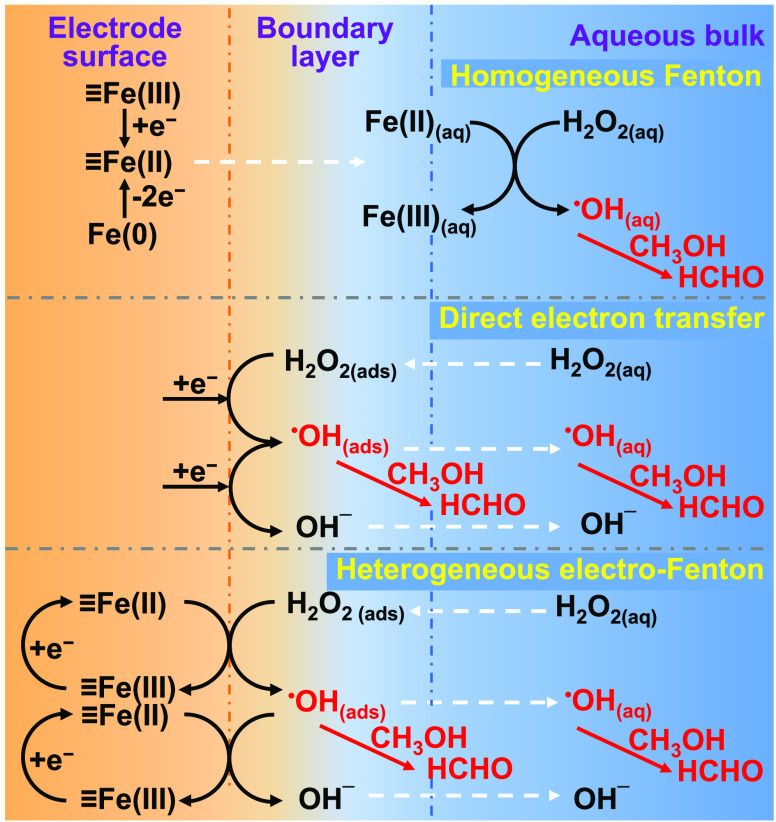
Possible mechanisms of activation of H_2_O_2_ into ^•^OH. The formation of Fe(IV) via the homogeneous Fenton pathway is not shown for the sake of simplicity.

H_2_O_2_ could potentially undergo three activation pathways to form ^•^OH: (1) homogeneous Fenton processes; (2) direct electron transfer reactions on the stainless-steel electrode; and (3) heterogeneous electro-Fenton processes (Figure 5). For the direct electron transfer and heterogeneous electro-Fenton pathways, any ^•^OH produced should initially consist of adsorbed species (^•^OH_ads_). These species can either react on the surface or diffuse into the aqueous phase. It should be noted that the reactivity of ^•^OH_ads_ toward organic compounds and its ability to diffuse into the aqueous phase are still unclear.^39^ The specific mechanism for formation of Fe(IV) species is beyond the scope of this study because Fe(IV) is less commonly used in water treatment due to its greater selectivity and low reactivity with contaminants.^10^

The homogeneous Fenton pathway for H_2_O_2_ activation was ruled out as an important source of contaminant-oxidizing species by predictions based on measured concentrations of dissolved Fe(II) during electrochemical H_2_O_2_ activation experiments. Homogeneous Fenton reactions would be expected to activate H_2_O_2_ at rates much lower than those observed at pH values of 4, 5, and 6 (Text S9). At pH values of 5 and above, this process should have produced mostly Fe(IV) instead of ^•^OH.^8,57,58^ Therefore, the production of ^•^OH by homogeneous Fenton processes can be excluded with respect to contaminant transformation at pH 5 and above. At pH 4, the homogeneous Fenton reaction accounted for about a third of the H_2_O_2_ loss in the solution phase.

Direct electron transfer from the stainless-steel electrode to H_2_O_2_ has been proposed as the mechanism through which H_2_O_2_ is converted into ^•^OH.^35^ The electrochemical reduction of H_2_O_2_ proceeds by one-electron-transfer processes at low overpotentials, such as those used in this study.^59−62^ In this process, H_2_O_2_ is first reduced to adsorbed ^•^OH_ads_. ^•^OH_ads_ is further reduced to H_2_O by a second electron. If this process takes place on the stainless-steel electrode, then ^•^OH_ads_ would have been produced as an intermediate. Diffusion of ^•^OH into solution and solutes capable of reacting with ^•^OH_ads_ would be in competition with the second electron transfer reaction on the electrode surface.

The heterogeneous electro-Fenton process can be initiated at +0.020 V and pH values below 7 by the reduction of ≡Fe(III) to ≡Fe(II). Surface ≡Fe(II) can react with H_2_O_2_ to produce ^•^OH_ads_.^63^ Following this reaction, ^•^OH_ads_ may react with ≡Fe(II) on the surface, resulting in the loss of the oxidizing species to the electrode surface. At pH values above 7 and a potential of +0.020 V, the electro-Fenton pathway was unlikely to occur because ≡Fe(III) (e.g., Fe_2_O_3_) is stable with respect to reduction.

Heterogeneous Fenton processes (i.e., reduction of ≡Fe(III) to ≡Fe(II) by H_2_O_2_ in the absence of an applied potential followed by reaction between ≡Fe(II) and H_2_O_2_ to produce ^•^OH) also could not account for H_2_O_2_ activation.^63^ Control experiments in which H_2_O_2_ was exposed to the stainless-steel electrode in the absence of an externally applied potential (Figure S19) indicated that activation of H_2_O_2_ through this mechanism was negligible relative to activation observed when a potential of +0.020 V was applied.

### Oxidation of Trace Organic Contaminants in Authentic Surface Water

The presence of natural organic matter and HCO_3_^–^ poses challenges for electrochemical advanced oxidation processes due to their ability to scavenge ^**•**^OH as well as their potential to cause fouling and scaling on the electrode surface.^64,65^ To assess the oxidation of trace organic contaminants under the optimal conditions identified previously (i.e., pH between 6 and 7 and an applied potential of +0.020 V), experiments were conducted in Na_2_SO_4_-amended surface water with the initial pH adjusted to 6.0. Because less than 0.05% of the produced ^**•**^OH was expected to react with the trace organic contaminants under conditions encountered in authentic surface water sample containing low concentrations of contaminants (i.e., 20 μg/L), a total of 15 mg/L of H_2_O_2_ was added to produce sufficient ^**•**^OH to transform contaminants in authentic surface waters (i.e., a small volume of concentrated H_2_O_2_ was added to obtain a concentration of 1.25 mg/L every 20 min throughout the treatment process, Figure S20). A slower rate of contaminant transformation would be expected at alkaline pH conditions due to the lower yield of ^**•**^OH from H_2_O_2_ activation. The extent at which system performance would decrease at higher pH values under realistic operating conditions could be important to reactor design and merits additional study. The presence of 200 mM Na_2_SO_4_ was not expected to affect the transformation of contaminants due to its inert chemical properties.^35,37^

**Figure 6:**
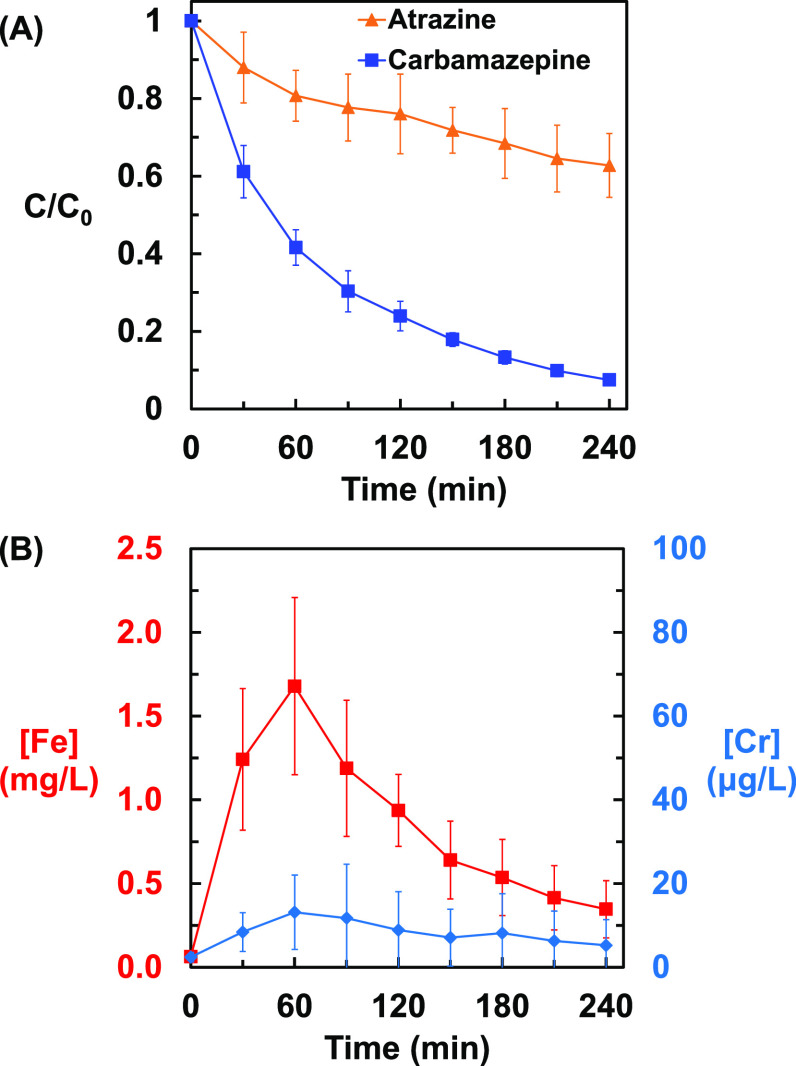
(A) Concentrations of representative trace organic contaminants and (B) concentrations of Fe and Cr released during the treatment of an authentic water sample. The authentic water sample was amended with 200 mM Na_2_SO_4_. Potential = +0.020 V, 1.25 mg/L of H_2_O_2_ was dosed every 20 min. Error bars represent one standard deviation.

Over 4 h of operation, more than 90% of the carbamazepine (*k*_carbamazepine,•OH_ = 9.1 × 10^9^ M^–1^ s^–1^)^66^ and about 40% of atrazine (*k*_Atrazine,•OH_= 2.5 × 10^9^ M^–1^ s^–1^)^67^ were removed (Figure 6A). The main reaction occurring at the Pt anode was expected to be oxygen evolution; the formation of reactive oxygen species on the anode that could contribute to probe compound oxidation was not expected to be important.^68^ Considering the total amount of H_2_O_2_ added during the experiment (i.e., 0.44 mM), we predict that approximately 0.3 mM ^**•**^OH was produced on the stainless-steel electrode surface. Based on the predicted fraction of the ^**•**^OH reacting with carbamazepine (0.03%) and atrazine (0.01%) under these conditions, transformation by ^**•**^OH was expected to remove more than 90% of carbamazepine and about 25% of atrazine, which was consistent with our observations. Model predictions also indicated that over 85% of the ^**•**^OH produced from H_2_O_2_ activation was lost on the electrode surface, and about 15% of the ^**•**^OH reacted with natural organic matter and other ^**•**^OH scavengers.

The maximum concentrations of Fe and Cr in the solution peaked at 60 min before decreasing to about 0.3 mg/L and 5 μg/L, respectively, at 240 min (Figure 6B). The decrease in Fe and Cr concentrations could have been caused by the precipitation of Fe(III)- and Cr(III)-oxide/(oxy)hydroxide at circumneutral pH conditions. The final concentration of leached Fe was near the secondary drinking water standard, and the final concentration of leached Cr in solution was well below the drinking water standard.^50,52^ If the electrodes had been preconditioned prior to starting the experiment (Text S1), it is likely that the observed concentrations of leached metals would have been substantially lower than those observed in this experiment and homogeneous Fenton process would have contributed much less to the overall transformation of contaminants.

## Environmental Implications

In comparison to the conventional approaches that assess electrode performance mainly by the observed transformation rate of a contaminant, this study provides an alternative approach for understanding the mechanisms contributing to process performance mechanistically by considering the production of reactive species and the fate of the reactive species in two separate processes. The low-cost stainless-steel electrode efficiently converted H_2_O_2_ into ^•^OH under circumneutral pH conditions. Nonetheless, most of the oxidant was lost to reactions on the electrode surface. Under conditions typical of drinking water (i.e., concentration of trace organic contaminant <0.1 μM and natural organic matter <10 mg of C/L), about 0.03% of the ^•^OH produced on the surface or in solution reacted with trace organic contaminants. Due to the dominant role of the surface as a sink for ^•^OH, the presence of natural organic matter had little effect on the treatment performance. Thus, under conditions encountered in many types of industrial wastewater or in highly colored drinking water sources, the performance of the stainless-steel electrode is not expected to be affected by dissolved species that compete for ^•^OH.

Despite the inherent inefficiency of the stainless-steel electrode, it may still be competitive with the UV/H_2_O_2_ process when used for a distributed water treatment. The equilibrium potential for the oxygen evolution reaction at the counter electrode at pH 7 is +0.817 V. Therefore, under the optimal potential of +0.020 V for the stainless-steel electrode, the theoretical minimum cell voltage will be around +0.8 V. Based on the electron utilization efficiency of 47% at pH 7 (Figure S12A), the minimum energy consumption to produce ^•^OH was estimated to be 0.05 kWh/mol. For comparison, a typical low-pressure UV lamp employed for UV/H_2_O_2_ treatment consumes about 0.37 kWh/mol to produce photons (electrical to UV conversion efficiency = 35%).^69^ Considering the inefficient absorption of UV light by H_2_O_2_ in natural waters (i.e., more than 95% of the produced UV light is absorbed by other chromophores),^5^ the energy consumption for generating ^•^OH by the UV/H_2_O_2_ process was estimated as 19 kWh/mol. Therefore, despite the loss of ^•^OH on the stainless-steel surface (∼90%), the energy consumption for producing ^•^OH that participated in the oxidation of aqueous species (i.e., 10 times the energy required to produce ^•^OH) was estimated as 0.5 kWh/mol, which is over an order of magnitude lower than that of point-of-use UV/H_2_O_2_ processes.

Furthermore, only about 10% of H_2_O_2_ that is added to the reactor is converted to ^•^OH in the UV/H_2_O_2_ process.^70,71^ Thus, the required H_2_O_2_ dosage, as well as the energy associated with H_2_O_2_ production for the UV/H_2_O_2_ process, will be about the same as in a system in which the same amount of ^•^OH that participates in the oxidation of aqueous species is produced by electrochemical activation on stainless-steel electrodes. Under many conditions, the stainless-steel electrode is expected to be more energy efficient than the UV/H_2_O_2_ process in terms of overall energy consumption (i.e., energy for H_2_O_2_ production and H_2_O_2_ activation).

In this study, the stainless-steel electrode was operated under batch conditions. However, the porous structure and the high surface area could enhance its kinetics when used in flow-through mode. Enhanced mass-transport of contaminants to the electrode surface could lead to higher contaminant concentrations near the electrode surface, thereby reducing the importance of the ^•^OH scavenging by the electrode surface. The replacement of the Pt counter electrode with other low-cost anodes (e.g., carbonaceous anodes) has a strong potential to further lower the cost of the system and enhance the rate of contaminant transformation through the production of anodically generated reactive species. Additional research is needed to assess this effect of flow-through conditions, as well as the long-term performance of the electrode. Research is also needed to develop reactor configurations that can operate at low ionic strength. Finally, the correlation between the material composition and the yield of ^•^OH as well as ^•^OH scavenging by the surface merits further investigation, especially in the presence of ions that affect the phases of Fe minerals present on the electrode (e.g., Si).

## Supporting Information Available

Details about the reactor schematic; precondition of the stainless-steel electrodes; analytical methods for analysis of H_2_O_2_, formaldehyde, acetone, trace organic contaminants, and metals; XPS analysis; electrolysis experiments in authentic surface water; effect of methanol concentration on H_2_O_2_ activation, current density, and metal leaching; effect of initial H_2_O_2_ concentration on H_2_O_2_ activation, current density, and metal leaching; kinetic predictions of H_2_O_2_ activation; thermodynamical analysis of the passivation layer on stainless-steel electrodes; electron utilization efficiency for producing reactive oxidants and for activating H_2_O_2_; and fate of ^•^OH for the treatment of trace organic contaminants under various concentrations of ^•^OH-scavenging compounds (PDF)
