# Supplementary material for: Surface Processes Control the Fate of Reactive Oxidants Generated by Electrochemical Activation of Hydrogen Peroxide on Stainless-Steel Electrodes
Source: Environ Sci Technol. 2023 Mar 16;57(47):18680–9. doi: 10.1021/acs.est.2c08404 (PMC10504418; doi:10.1021/acs.est.2c08404)
Supplement: Supplementary file 1 — es2c08404_si_001.pdf [file es2c08404_si_001.pdf]

Supplementary Material for:

Surface Processes Control the Fate of Reactive Oxidants Generated by Electrochemical Activation of Hydrogen Peroxide on Stainless-Steel Electrodes

Yanghua Duan, Wenli Jiang, and David L. Sedlak*

Department of Civil & Environmental Engineering

University of California, Berkeley, CA, 94720

29 pages

3 tables

20 figures

References

*Corresponding author e-mail: sedlak@berkeley.edu, T: 510-643-0256

Table of Contents

Figure S1. Schematic configuration of the electrochemical reactor. The system was operated at a fixed potential. Both chambers were stirred with Teflon-coated magnetic stir bars at 400 rotations per minute. S5

Text S1. Pre-condition of the stainless-steel electrode........ S5

Figure S2. (A) H_2O_2 activation, (B) formaldehyde formation, (C-E) metal leaching, and (F) current density during the pre-conditioning processes. Potential = +0.020 V when not specified. Error bars and confidence interval bands represent one standard deviation........ S7

Figure S3. H_2O_2 concentrations in the presence of 100 mM of (A) methanol, (B) 2-propanol in buffer-containing Na_2SO_4 electrolyte. The experiments were conducted in the absence of stainless-steel electrodes. No formation of formaldehyde or acetone was observed. Error bars represent one standard deviation........ S8

Figure S4. H_2O_2 concentrations in the cathode chamber at (A) pH 6 and (B) pH 7 with various concentrations of methanol in electrolyte. (C) Current density as a function of methanol concentration. Potential = +0.020V, $[H_2O_2]_0$ = 1.25 mg/L. Error bars represent one standard deviation; error bars not shown are smaller than symbols........ S9

Text S2. Electrolysis in authentic surface water S10

Figure S5. Schematic configuration of the undivided electrochemical reactor. S10

Table S1. Water quality parameters of the surface water. S11

Figure S6. Concentrations of (A) H_2O_2 and (B) trace organic contaminants in Na_2SO_4-amended surface water in the absence of stainless-steel electrode. $[H_2O_2]_0$ = 1.25 mg/L. Error bars represent one standard deviation; error bars not shown are smaller than symbols........ S12

Figure S7. Concentrations of trace organic contaminants in Na_2SO_4-amended surface water in the absence of H_2O_2. Potential = +0.020 V. Error bars represent one standard deviation; error bars not shown are smaller than symbols. S12

Text S3. Analytical methods S13

Figure S8. H_2O_2 concentrations in the cathode chamber. Potential = +0.020V when not specified, $[H_2O_2]_0$ = 1.25 mg/L. [Probe compound] = 100 mM. Error bars represent one standard deviation; error bars not shown are smaller than symbols........ S14

Figure S9. Pourbaix diagram for (A) Fe, $[Fe]_{tot}$ = 1 mg/L, (B) Cr, $[Cr]_{tot}$ = 10 μg/L and (C) H_2O_2, $[H_2O_2]$ = 37 μM and $[^{\bullet}OH]_{SS} = 3.6 \times 10^{-11}$ M. The $[^{\bullet}OH]_{SS}$ was estimated based on a previously reported 1,4-dioxane degradation kinetics under similar operating conditions.[7] Symbols represent experimental conditions tested in this study. S14

Table S2. Reactions considered for construction of Pourbaix diagrams........ S15

Figure S10. H_2O_2 activation in the cathode chamber at varying initial H_2O_2 concentration. Applied potential = +0.020V, pH = 6. .... S16

Text S4. Prediction of the reaction rate constant based on mass-transport limitation S16

Figure S11. (A) Current densities observed under different experimental conditions. (B) Relationship between the observed current densities and initial H_2O_2 concentration. Applied potential = +0.020 V, pH = 6. Experiments conducted in deareated solution was purged with N_2 for at least 20 minutes before the experiment and was continuously purged with N_2 throughout the experiments in the sealed H-cell reactor. The flow rate of the N_2 stream was maintained at 0.5 L/min. Error bars represent one standard deviation. S17

Figure S12. Electron utilization efficiency for (A) producing reactive oxidants and (B) for activating H_2O_2 as a function of pH and applied potential. $[H_2O_2]_0$ = 1.25 mg/L, [Probe compound] = 100 mM. Error bars represent one standard deviation; error bars not shown are smaller than symbols. S18

Figure S13. Concentrations of (A) total Fe, (B) Fe(II), (C) total Cr, and (D) Cr(VI) leached from the stainless steel electrodes at different pH and potentials. Potential = + 0.020 V when not specified. Error bars represent one standard deviation. S19

Figure S14. Atomic concentration of Fe in different oxidation states. Potential = +0.020 V, The * symbol represents experiments conducted at potentials lower than +0.020 V (i.e., -0.039 V at pH 8 and -0.098 V at pH9). Error bars represent one standard deviation. S19

Figure S15. Metal concentrations after five minutes of electrolysis under various experimental conditions. Potential = +0.020V, pH = 6. Error bars represent one standard deviation. Experiments conducted in deareated solution was purged with N_2 for at least 20 minutes before the experiment and was continuously purged with N_2 throughout the experiments in the sealed H-cell reactor. The flow rate of the N_2 stream was maintained at 0.5 L/min. Error bars represent one standard deviation. S20

Figure S16. Metal concentrations during five minutes of electrolysis of electrolyte containing varying concentrations of methanol. Potential = +0.020 V. Error bars represent one standard deviation. S21

Text S5. Reaction rate constant for the reaction between $^{\bullet}OH$ and the electrode surface S21

Figure S17. Linear relationship between inverse of formaldehyde yield and inverse of concentration of methanol. Error bars represent one standard deviation. S22

Text S6. Prediction of the fate of $^{\bullet}OH$. S22

Text S7. Reaction rate constants for $^{\bullet}OH$ with organic buffer compounds. S23

Figure S18. Natural logarithm of normalized concentration versus time for carbamazepine and (A) MES in the presence of H_2O_2 at pH 6, (B) PIPES in the presence of H_2O_2 at pH 7, (C) MES in the absence of H_2O_2 at pH 6 and (D) PIPES in the absence of H_2O_2 at pH 7. [Test

compound]$_{init.}$ = 1.0 μM, [carbamazepine]$_{init.}$ = 0.5 μM, [H_2O_2]$_{init.}$ = 10 μM, [PO_4^{3-}] = 100 μM. Error bars represent one standard deviation....... S24

Text S8. Analytical methods for detection of MES and PIPES S24

Table S3. Compound-specific mass spectroscopy parameters....... S25

Text S9. Prediction of reaction rates between H_2O_2 and Fe(II). S26

Figure S19. H_2O_2 activation and formaldehyde formation at open circuit potential and +0.020 V. Experiments conducted in buffered Na_2SO_4 electrolyte, pH = 6. [H_2O_2]$_0$ = 1.25 mg/L. Error bars represent one standard deviation....... S26

Figure S20. (A) H_2O_2 concentrations and (B) observed current densities during the treatment of an authentic water sample. Potential = +0.020 V, 1.25 mg/L of H_2O_2 was dosed every 20 minutes. Error bars represent one standard deviation....... S27

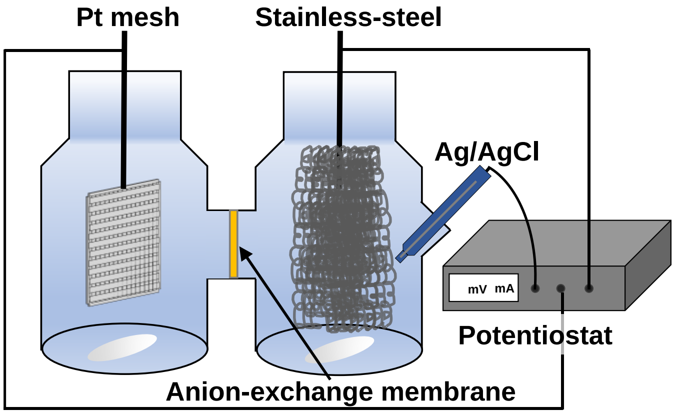

Figure S1. Schematic configuration of the electrochemical reactor. The system was operated at a fixed potential. Both chambers were stirred with Teflon-coated magnetic stir bars at 400 rotations per minute.

Text S1. Pre-condition of the stainless-steel electrode

To provide a stable performance, a new Scotch-Brite 20 g stainless-steel scrubber (catalogue number 214C, 3M Company, St. Paul, MN, USA; 80 cm^2/g specific surface area) was cut to around 4.5 g and pre-conditioned for each operating condition tested (i.e., pH and potential). The same electrode was used for all experiments under that operating condition. No significant decrease in the yield of oxidants was observed over time except in experiments conducted at pH 6 and +0.020 V. In these experiments, decreases in performance were observed after pre-conditioning and after completion of 15 experiments. After the decreases in performance were observed, another electrode was pre-conditioned following the same protocol and used for the remaining experiments. Before and after each experiment, the stainless-steel electrodes were rinsed thoroughly with ultrapure water and air-dried for at least 30 minutes before being reused.

The pre-conditioning process was performed with the working electrode chamber operated in continuous stirred-tank reactor mode to avoid any metal accumulation. The working electrode chamber was fed with a 0.2 M Na_2SO_4 electrolyte that containing methanol (100 mM),

buffer and H_2O_2 as described in the Materials and Methods Section. A total of about 5 L of electrolyte was fed into the working electrode chamber over one hour. Hydraulic residence time was maintained at 1.20 ± 0.03 min.

During the pre-conditioning process, new stainless-steel electrodes underwent a period of rapid corrosion which was accompanied by the release of metals and variation in performance. After approximately one hour, the leaching of metals substantially decreased and the kinetics of H_2O_2 activation became more reproducible. When operated at pH values below 7 and a potential of +0.020 V in continuous flow mode, elevated metal concentrations were observed within 20 minutes (Figure S2C-E). At higher pH values, substantial metal leaching was never observed. Therefore, pre-conditioning (i.e., operating the electrode for one hour) followed by disposal of leached metals may be needed prior to treating water with an initial pH value below 7. Under all tested conditions, the electrode performance stabilized within one hour in terms of H_2O_2 activation, reactive oxidant production, metal leaching, and current density (Figure S2).

125

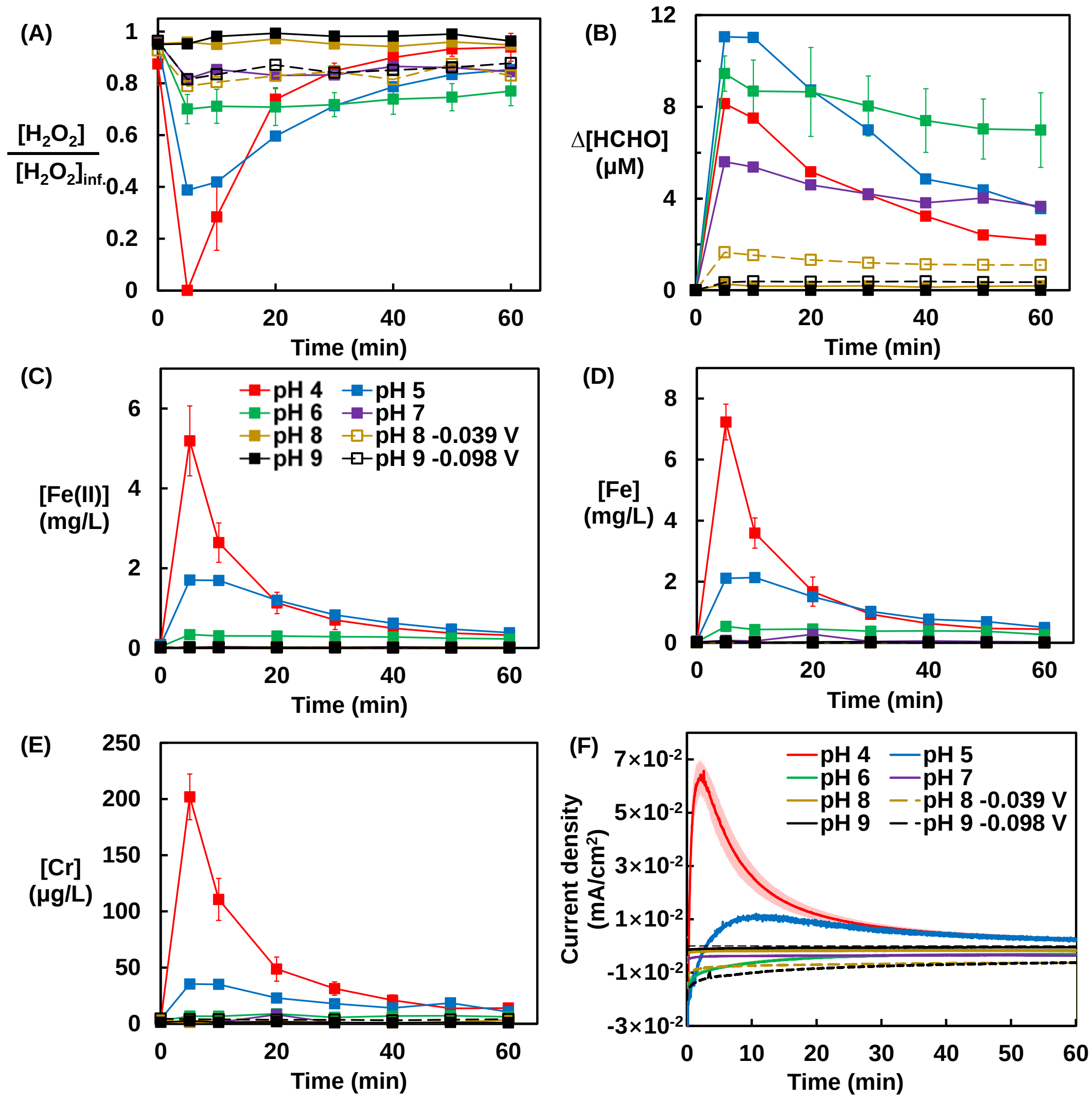

126 **Figure S2.** (A) H_2O_2 activation, (B) formaldehyde formation, (C-E) metal leaching, and (F)
127 current density during the pre-conditioning processes. Potential = +0.020 V when not specified.
128 Error bars and confidence interval bands represent one standard deviation.

129

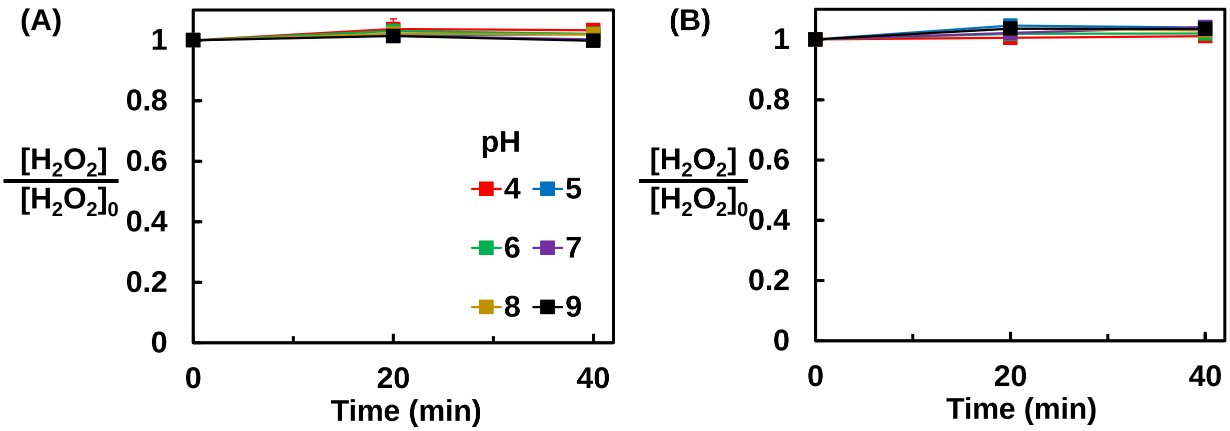

Figure S3. H_2O_2 concentrations in the presence of 100 mM of (A) methanol, (B) 2-propanol in buffer-containing Na_2SO_4 electrolyte. The experiments were conducted in the absence of stainless-steel electrodes. No formation of formaldehyde or acetone was observed. Error bars represent one standard deviation.

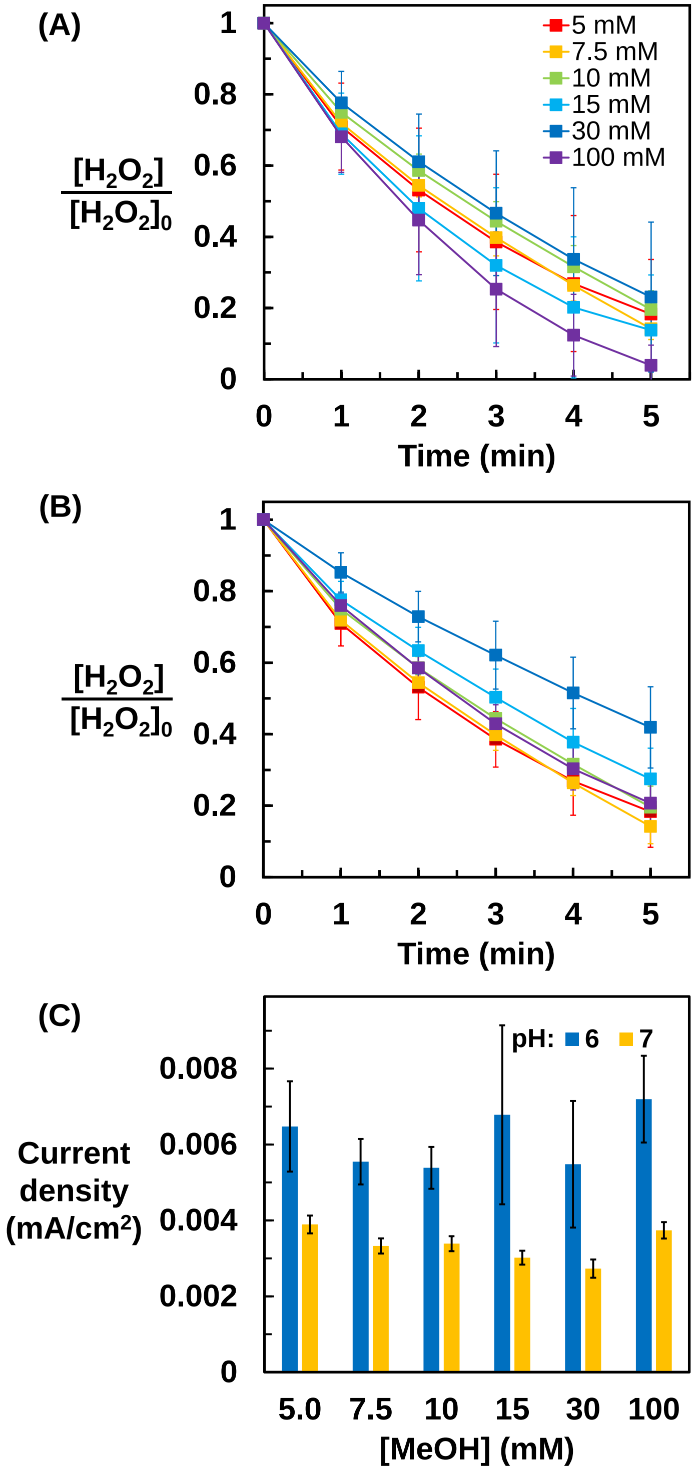

Figure S4. H_2O_2 concentrations in the cathode chamber at (A) pH 6 and (B) pH 7 with various concentrations of methanol in electrolyte. (C) Current density as a function of methanol concentration. Potential = +0.020V, $[H_2O_2]_0$ = 1.25 mg/L. Error bars represent one standard deviation; error bars not shown are smaller than symbols.

Text S2. Electrolysis in authentic surface water

Electrolysis experiments were performed in an undivided reactor (Figure S5, V = 125 mL) stirred at 600 rotations per minute with a Teflon stir bar. A fresh stainless-steel electrode (~4.5g) was used for each replicate. Because of the long duration of the electrolysis experiment (i.e., four hours), the pre-conditioning process was included in the experiments. H_2O_2 (1.25 mg/L = 37 μM) was dosed every 20 minutes throughout the electrolysis experiments.

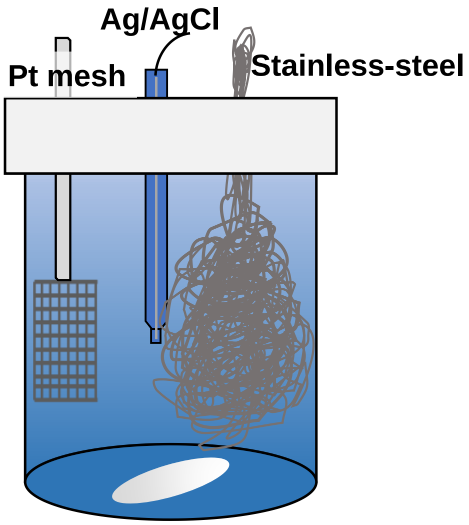

Figure S5. Schematic configuration of the undivided electrochemical reactor.

Surface water was collected during a storm event from Strawberry Creek on the University of California, Berkeley campus (37°52'26.9"N 122°15'41.8"W) on December 22, 2021, around 1:30 PM, Pacific Time. The surface water runoff was filtered through 0.7 µm glass fiber filter (MilliporeSigma, Burlington, MA) followed by 0.45 µm Supor® 450 Membrane (Gelman Sciences, Ann Arbor, MI) prior to experiment. A summary of the water quality parameters is provided in Table S1.

Table S1. Water quality parameters of the surface water.

Parameter	Value
Alkalinity	2.07±0.02 mM
Dissolved organic carbon	5.1±0.1 mg-C/L
$SUVA_{254}$	0.034±0.001 L mg-C^{-1} cm^{-1}
Conductivity	255 μS/cm
pH	7.83±0.04
F^-	0.14 mg/L
Cl^-	10.0 mg/L
Br^-	< 0.58 mg/L
NO_2^-	< 0.78 mg/L
NO_3^-	2.70 mg/L
PO_4^{3-}	< 2.0 mg/L
SO_4^{2-}	16.9 mg/L
Li^+	< 0.14 mg/L
Na^+	15.8 mg/L
K^+	< 1.0 mg/L
NH_4^+	< 2.1 mg/L
Mg^{2+}	8.07 mg/L
Ca^{2+}	21.5 mg/L
Total Si	< 1.2 mg/L

The collected surface water was amended with 200 mM of Na_2SO_4 to avoid overloading of the potentiostat. To test the performance of the stainless-steel electrode under circumneutral pH conditions, the initial pH of the surface water was adjusted from 7.8 to 6.0 with diluted H_2SO_4 prior to the electrolysis. (Solution acidification on this magnitude might also occur if the electrode had been part of a three-electrode system in which the cathodes for H_2O_2 production and activation were preceded by an anode.) The pH of the solution increased from 6.0 to around 7.3 gradually during electrolysis, potentially caused by CO_2 partitioning between the solution and atmosphere. Based on its alkalinity, the equilibrium pH was estimated to be 8.0 when the water was equilibrated with the atmosphere.

Control experiments indicated that H_2O_2 did not react with carbamazepine or atrazine in the absence of stainless-steel electrode (Figure S6).

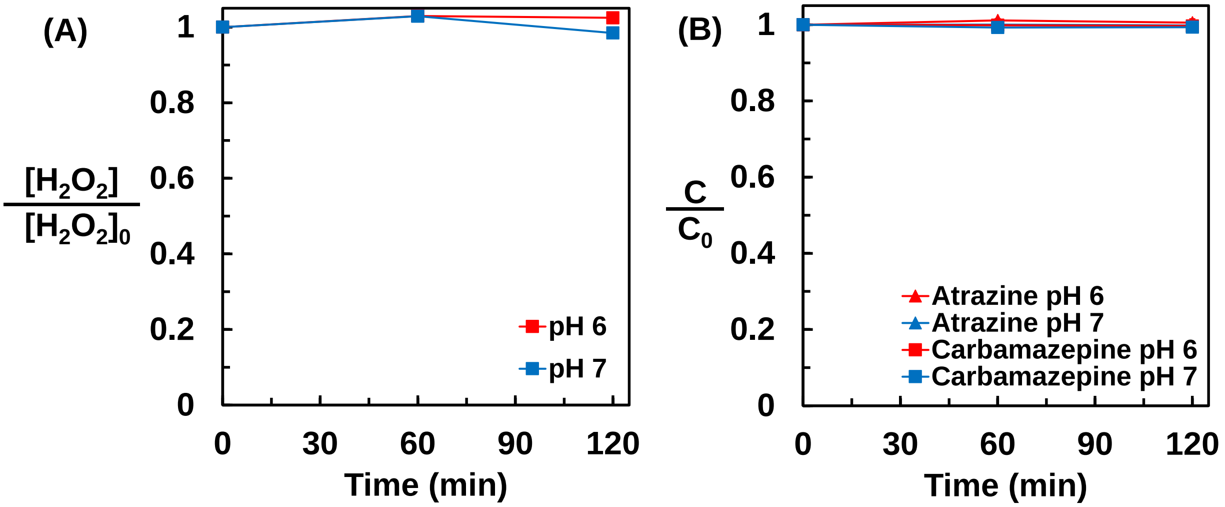

Figure S6. Concentrations of (A) H_2O_2 and (B) trace organic contaminants in Na_2SO_4-amended surface water in the absence of stainless-steel electrode. $[H_2O_2]_0$ = 1.25 mg/L. Error bars represent one standard deviation; error bars not shown are smaller than symbols.

Control experiments were also conducted in the absence of H_2O_2 to assess contaminant removal by processes taking place on the electrodes (e.g., direct electron transfer). Results indicated less than 10% loss of atrazine and about 25% loss of carbamazepine over the two-hour experiment (Figure S7). This was substantially lower than the removal observed in the presence of H_2O_2 (Figure 6A).

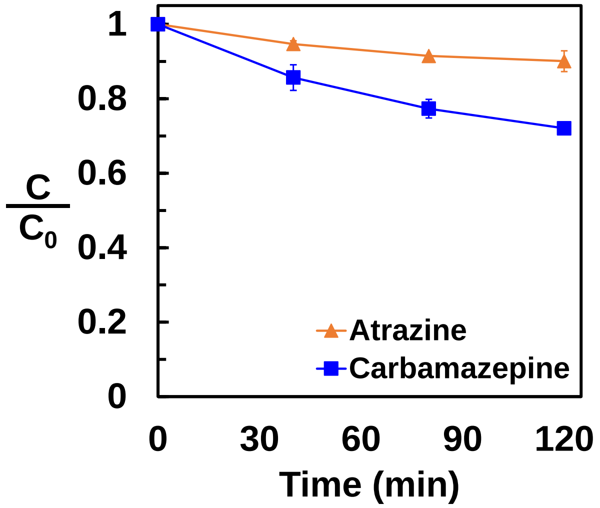

Figure S7. Concentrations of trace organic contaminants in Na_2SO_4-amended surface water in the absence of H_2O_2. Potential = +0.020 V. Error bars represent one standard deviation; error bars not shown are smaller than symbols.

Text S3. Analytical methods

To prevent continued formation of formaldehyde or acetone from reactions between residual H_2O_2 and Fe(II) remaining at the time of collection, an aliquot of 1,10-phenanthroline (final concentration = 1 mM) was added to the samples.[1] Formaldehyde and acetone were analyzed using 2,4-dinitrophenylhydrazine (DNPH) derivatization followed by analysis on an Agilent 1260 Infinity high-performance liquid chromatography (HPLC) equipped with diode array detector as described previously.[2]

Samples for H_2O_2 and Fe(II) measurement were quenched by mixing with colorimetric reagents immediately after collection. H_2O_2 was measured by a modified version of the peroxidase catalyzed N,N-diethyl-p-phenylenediamine (DPD) oxidation method.[3] Fe(II) was measured with a modified version of the ferrozine method with addition of an aliquot of ammonia fluoride (final concentration = 7 mM) to prevent interference from Fe(III).[2, 4] Cr(VI) was measured with the diphenylcarbazide method.[5] Aliquots for total metals measurement were acidified immediately after sampling to prevent precipitation and quantified in triplicate on an Agilent 7700 Series Inductively Coupled Plasma-Mass Spectrometer (ICP-MS).

Methanol (10% v/v) was added to carbamazepine- and atrazine-containing samples to quench any possible reactions. Carbamazepine and atrazine were quantified in multiple reaction monitoring (MRM) mode with an Agilent 1260 series HPLC system coupled to a 6460 triple quadrupole tandem mass spectrometer (HPLC-MS/MS) as previously described.[6]

The oxidation state of Fe in the surface of stainless-steel electrodes was characterized by X-ray photoelectron spectroscopy with a K-Alpha XPS spectrometer (Thermo Scientific Ltd., East Grinstead, UK). Samples were air-dried prior to characterization.

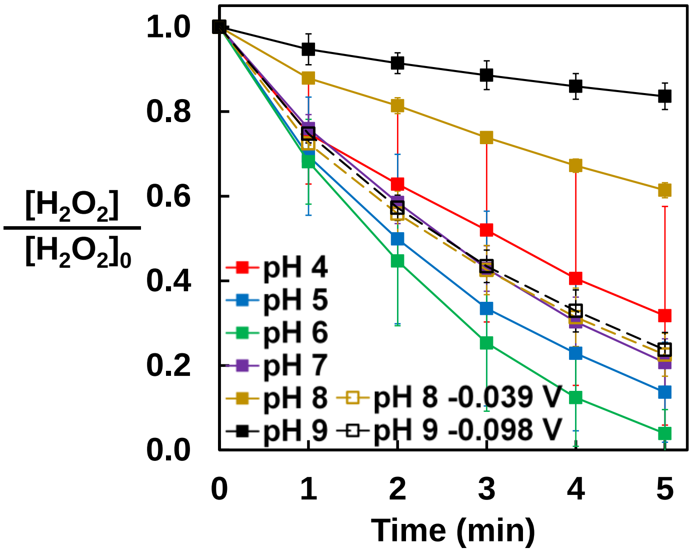

Figure S8. H_2O_2 concentrations in the cathode chamber. Potential = +0.020V when not specified, $[H_2O_2]_0$ = 1.25 mg/L. [Probe compound] = 100 mM. Error bars represent one standard deviation; error bars not shown are smaller than symbols.

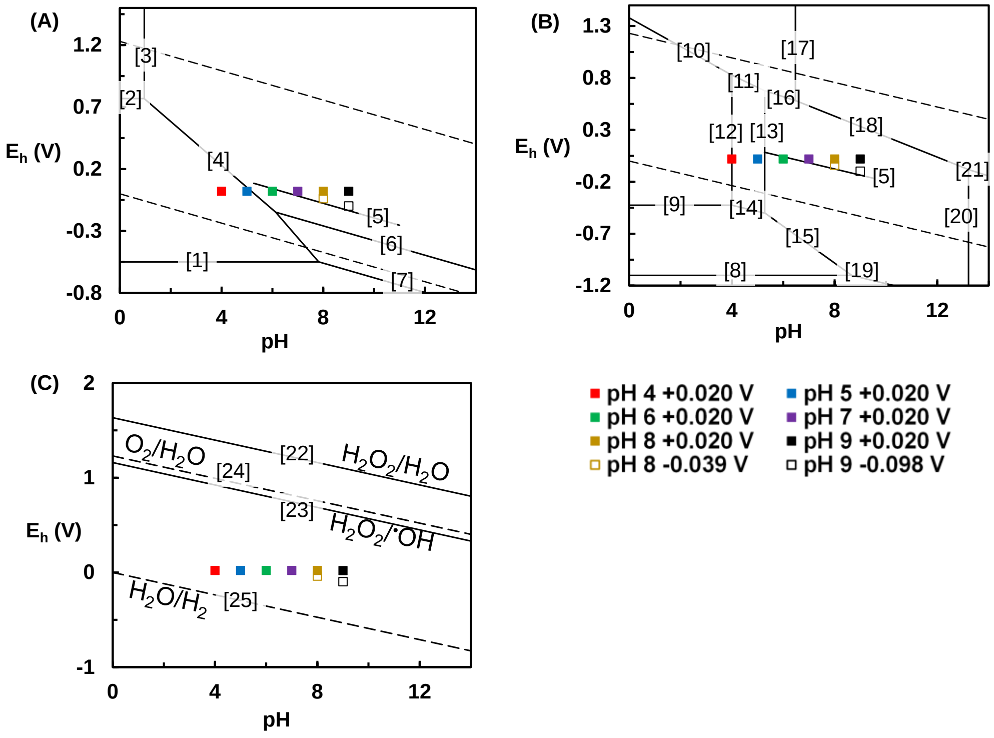

Figure S9. Pourbaix diagram for (A) Fe, $[Fe]_{tot}$ = 1 mg/L, (B) Cr, $[Cr]_{tot}$ = 10 μg/L and (C) H_2O_2, $[H_2O_2]$ = 37 μM and $[^{\bullet}OH]_{SS} = 3.6 \times 10^{-11}$ M. The $[^{\bullet}OH]_{SS}$ was estimated based on a previously reported 1,4-dioxane degradation kinetics under similar operating conditions.[7] Symbols represent experimental conditions tested in this study.

Table S2. Reactions considered for construction of Pourbaix diagrams.

[1]	$Fe \leftrightarrow Fe^{2+} + 2e^{-}$	E° = -0.41 V
[2]	$Fe^{2+} \leftrightarrow Fe^{3+} + e^{-}$	E° = +0.77 V
[3]	$2Fe^{3+} + 3H_2O \leftrightarrow Fe_2O_3 + 6H^{+}$	$K = 10^{3.73}$
[4]	$2Fe^{2+} + 3H_2O \leftrightarrow Fe_2O_3 + 6H^{+} + 2e^{-}$	E° = +0.66 V
[5]	$2FeCr_2O_4 + H_2O \leftrightarrow Fe_2O_3 + 2Cr_2O_3 + 2H^{+} + 2e^{-}$	E° = +0.40 V
[6]	$2Fe_3O_4 + H_2O \leftrightarrow 3Fe_2O_3 + 2H^{+} + 2e^{-}$	E° = +0.21 V
[7]	$3Fe + 4H_2O \leftrightarrow Fe_3O_4 + 8H^{+} + 8e^{-}$	E° = -0.09 V
[8]	$Cr \leftrightarrow Cr^{2+} + 2e^{-}$	E° = -0.90 V
[9]	$Cr^{2+} \leftrightarrow Cr^{3+} + e^{-}$	E° = -0.43 V
[10]	$Cr^{3+} + 4H_2O \leftrightarrow HCrO_4^{-} + 3e^{-} + 7H^{+}$	E° = +1.38 V
[11]	$CrOH^{2+} + 3H_2O \leftrightarrow HCrO_4^{-} + 3e^{-} + 6H^{+}$	E° = +1.30 V
[12]	$Cr^{3+} + H_2O \leftrightarrow CrOH^{2+} + H^{+}$	$K = 10^{-4.00}$
[13]	$2CrOH^{2+} + H_2O \leftrightarrow Cr_2O_3 + 4H^{+}$	$K = 10^{-7.68}$
[14]	$Cr^{2+} + H_2O \leftrightarrow CrOH^{2+} + H^{+} + e^{-}$	E° = -0.19 V
[15]	$2Cr^{2+} + 3H_2O \leftrightarrow Cr_2O_3 + 6H^{+} + 2e^{-}$	E° = +0.04 V
[16]	$Cr_2O_3 + 5H_2O \leftrightarrow 2HCrO_4^{-} + 6e^{-} + 8H^{+}$	E° = 1.23 V
[17]	$HCrO_4^{-} \leftrightarrow CrO_4^{2-} + H^{+}$	$K = 10^{-6.47}$
[18]	$Cr_2O_3 + 5H_2O \leftrightarrow 2CrO_4^{2-} + 6e^{-} + 10H^{+}$	E° = 1.35 V
[19]	$2Cr + 3H_2O \leftrightarrow Cr_2O_3 + 6e^{-} + 6H^{+}$	E° = -0.59 V
[20]	$Cr_2O_3 + 5H_2O \leftrightarrow 2Cr(OH)_4^{-} + 2H^{+}$	$K = 10^{-39.86}$
[21]	$Cr(OH)_4^{-} \leftrightarrow CrO_4^{2-} + 3e^{-} + 4H^{+}$	E° = 0.96 V
[22]	$H_2O_2 + 2e^{-} + 2H^{+} \rightarrow 2H_2O$	E° = 1.76 V
[23]	$H_2O_2 + e^{-} + H^{+} \rightarrow H_2O + {}^{\bullet}OH$	E° = 0.80 V
[24]	$2H_2O \leftrightarrow O_2 + 4e^{-} + 4H^{+}$	E° = 1.23 V
[25]	$H_2 \rightarrow 2e^{-} + 2H^{+}$	E° = 0.00 V
[26]	$Fe(OH)_2 \rightarrow Fe^{2+} + 2OH^{-}$	$K = 10^{-16.32}$

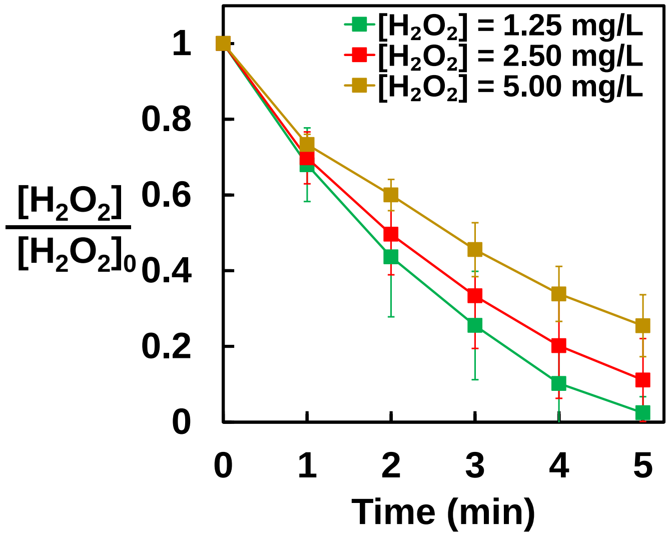

Figure S10. H_2O_2 activation in the cathode chamber at varying initial H_2O_2 concentration. Applied potential = +0.020V, pH = 6.

Text S4. Prediction of the reaction rate constant based on mass-transport limitation

The mass-transport limiting current density was predicted based on film theory. The mass-transfer coefficient k_m was predicted by:

$$k_{\mathrm{m}} = \frac{D}{L_{\mathrm{f}}} \qquad \text{(S1)}$$

where D is the diffusion coefficient of H_2O_2 in water (1.5×10^{-5} cm^2 s^{-1}),[8, 9] and L_f is the film thickness (about 100 μm).[10, 11]

The rate constant limited by mass transport, k_{limt}, was predicted by:

$$k_{\mathrm{limt}} = k_{\mathrm{m}} a \qquad \text{(S2)}$$

where a is the surface area to volume ratio (m^2/m^3).

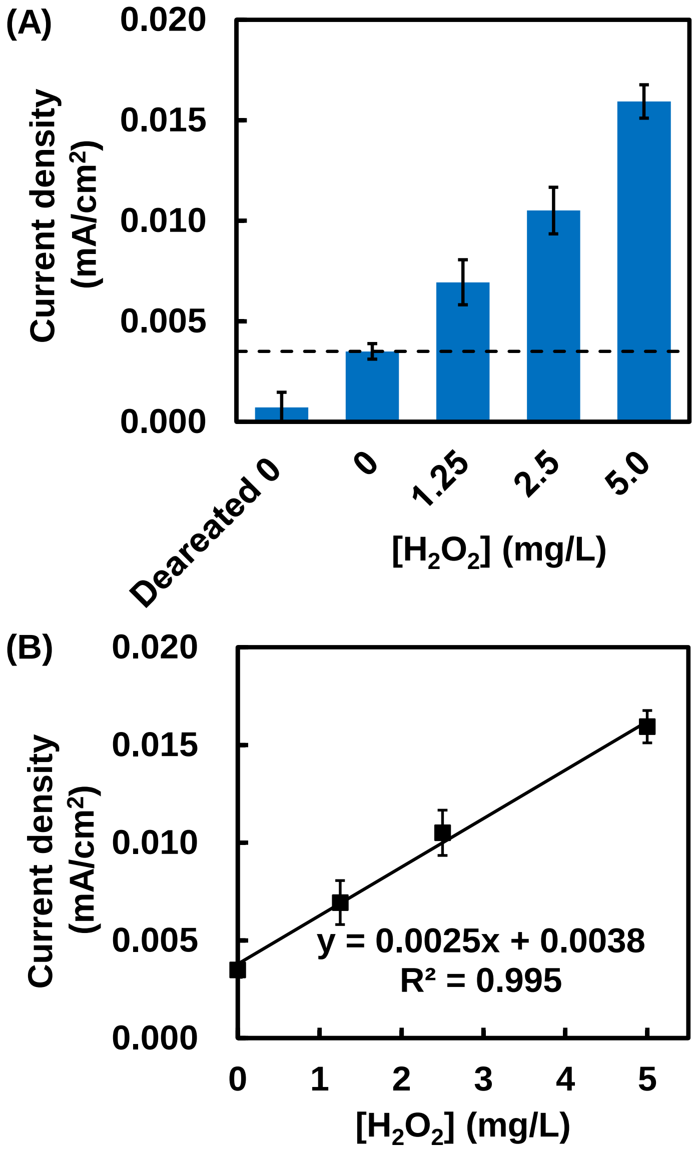

Figure S11. (A) Current densities observed under different experimental conditions. (B) Relationship between the observed current densities and initial H_2O_2 concentration. Applied potential = +0.020 V, pH = 6. Experiments conducted in deareated solution was purged with N_2 for at least 20 minutes before the experiment and was continuously purged with N_2 throughout the experiments in the sealed H-cell reactor. The flow rate of the N_2 stream was maintained at 0.5 L/min. Error bars represent one standard deviation.

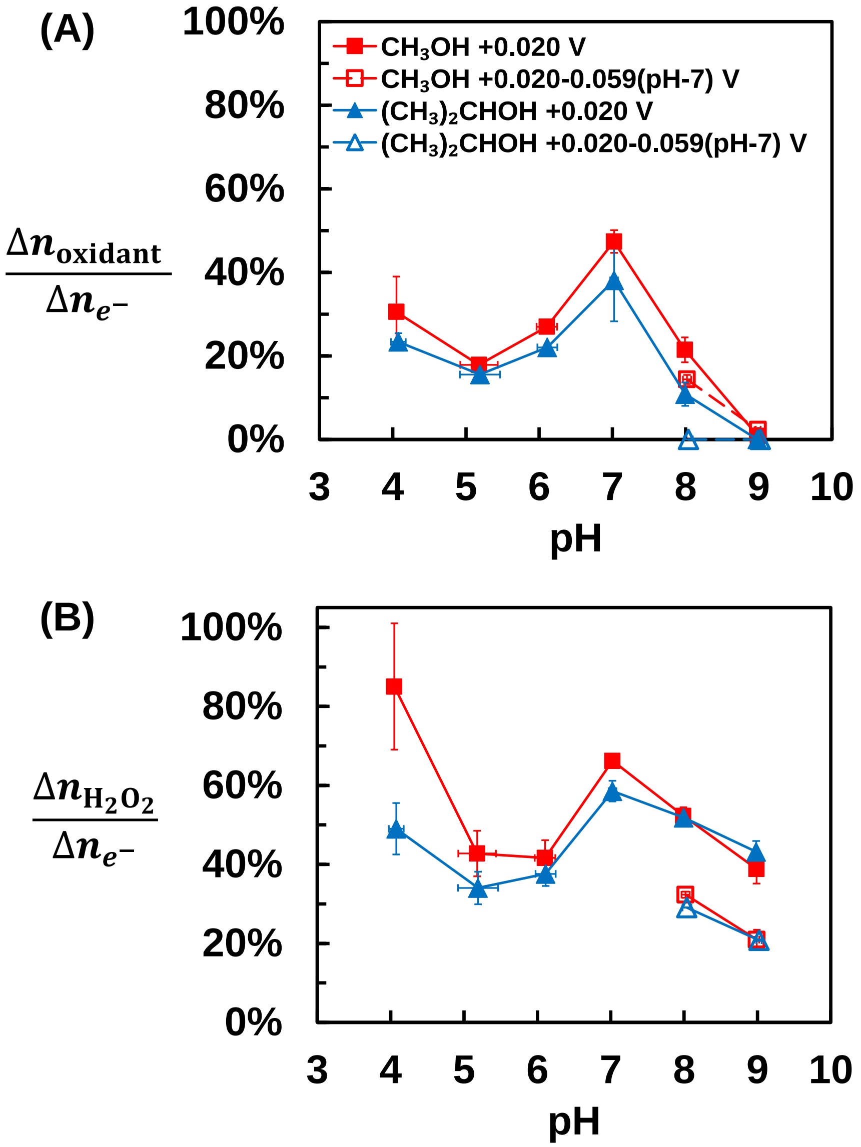

Figure S12. Electron utilization efficiency for (A) producing reactive oxidants and (B) for activating H_2O_2 as a function of pH and applied potential. $[H_2O_2]_0$ = 1.25 mg/L, [Probe compound] = 100 mM. Error bars represent one standard deviation; error bars not shown are smaller than symbols.

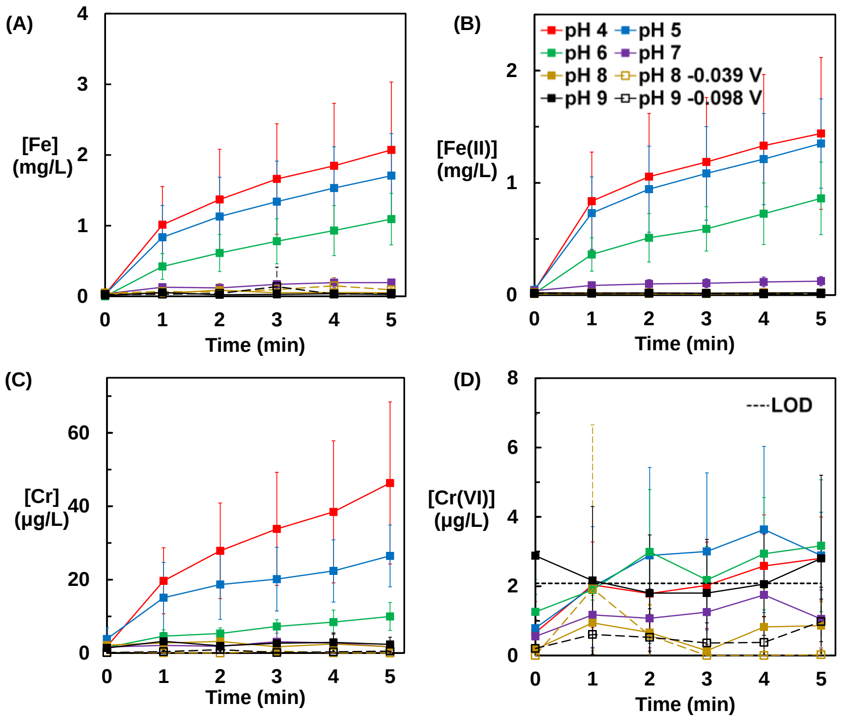

Figure S13. Concentrations of (A) total Fe, (B) Fe(II), (C) total Cr, and (D) Cr(VI) leached from the stainless steel electrodes at different pH and potentials. Potential = + 0.020 V when not specified. Error bars represent one standard deviation.

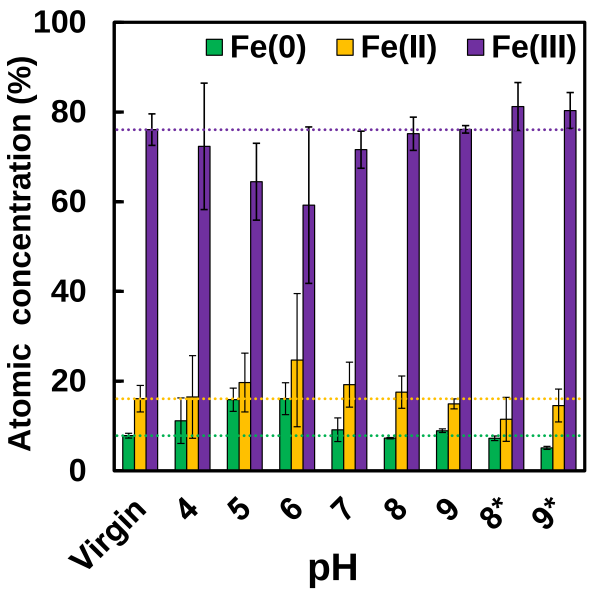

Figure S14. Atomic concentration of Fe in different oxidation states. Potential = +0.020 V, The * symbol represents experiments conducted at potentials lower than +0.020 V (i.e., -0.039 V at pH 8 and -0.098 V at pH9). Error bars represent one standard deviation.

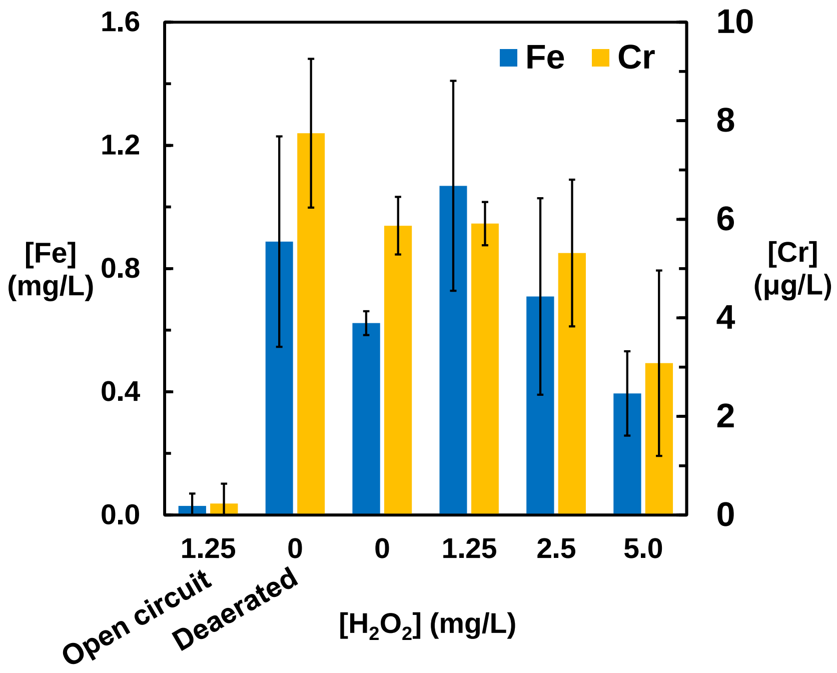

Figure S15. Metal concentrations after five minutes of electrolysis under various experimental conditions. Potential = +0.020V, pH = 6. Error bars represent one standard deviation. Experiments conducted in deareated solution was purged with N_2 for at least 20 minutes before the experiment and was continuously purged with N_2 throughout the experiments in the sealed H-cell reactor. The flow rate of the N_2 stream was maintained at 0.5 L/min. Error bars represent one standard deviation.

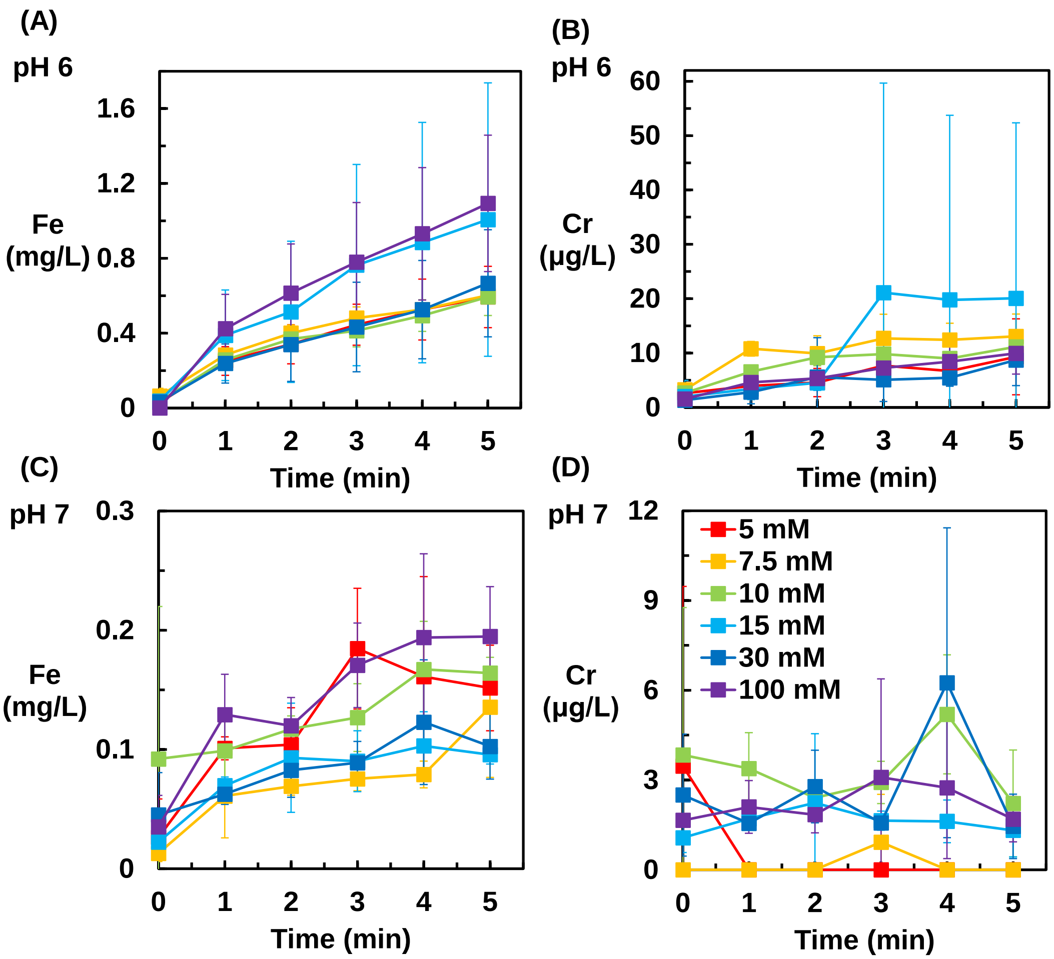

Figure S16. Metal concentrations during five minutes of electrolysis of electrolyte containing varying concentrations of methanol. Potential = +0.020 V. Error bars represent one standard deviation.

Text S5. Reaction rate constant for the reaction between $^{\bullet}$OH and the electrode surface

The reaction rate constant for the reaction between $^{\bullet}$OH and the electrode surface $k_{\text{surface},\bullet\text{OH}}$ was calculated by rearranging eq.3 into a linear form (eq.S3, Figure S17) and subtracting out contributions from H_2O_2, Fe^{2+} and the buffer to $^{\bullet}$OH scavenging.

$$\frac{1}{\text{HCHO yield}} = \frac{(k_{\text{H}_2\text{O}_2,\bullet\text{OH}}[\text{H}_2\text{O}_2]+k_{\text{Buffer},\bullet\text{OH}}[\text{Buffer}]+k_{\text{surface},\bullet\text{OH}}[\text{Surface}]+k_{\text{Fe}^{2+},\bullet\text{OH}}[\text{Fe}^{2+}])}{\bullet\text{OH yield } k_{\text{CH}_3\text{OH},\bullet\text{OH}}}\frac{1}{[\text{CH}_3\text{OH}]}+\frac{1}{\bullet\text{OH yield}} \quad \text{(S3)}$$

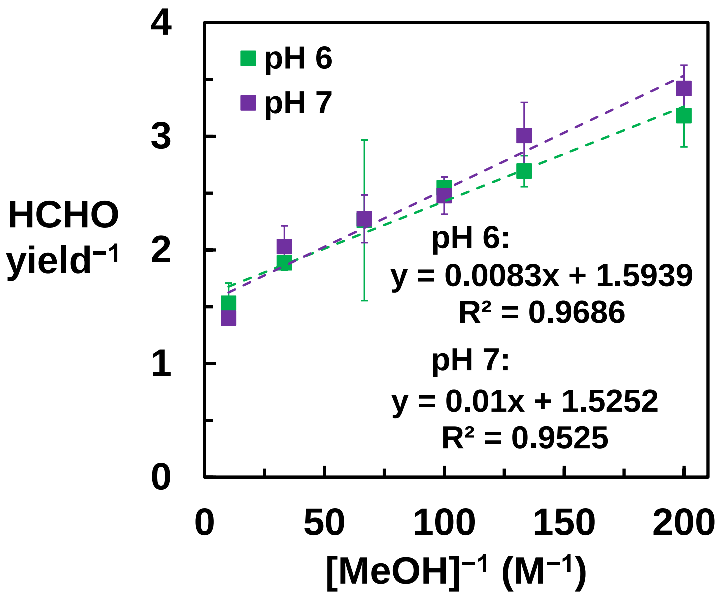

Figure S17. Linear relationship between inverse of formaldehyde yield and inverse of concentration of methanol. Error bars represent one standard deviation.

The second-order rate constants for the reactions of buffers with $^{\bullet}$OH were measured using competition kinetics as $k_{\mathrm{MES},\bullet\mathrm{OH}}$ = 2.1±0.1 ×10^{9} M^{-1} s^{-1} and $k_{\mathrm{PIPES},\bullet\mathrm{OH}}$ = 4.2±0.1×10^{9} M^{-1} s^{-1}, respectively (Text S7). The reaction rate constant for the reaction between the electrode surface and $^{\bullet}$OH was calculated as 6.5 × 10^{5} g^{-1} s^{-1} at pH 6 and 4.9 × 10^{5} g^{-1} s^{-1} at pH 7.

Text S6. Prediction of the fate of $^{\bullet}$OH.

The fraction of $^{\bullet}$OH that reacted with the electrode surface and aqueous species was estimated by:

$$\text{Fraction of } {}^{\bullet}\text{OH to species } i = \frac{k_{\bullet\mathrm{OH},i}[i]}{\sum k_{\bullet\mathrm{OH},j}[j]} \qquad \text{(S4)}$$

where *j* represents species that reacts with $^{\bullet}$OH (e.g., electrode surface, competing organic compounds, Fe^{2+}, H_2O_2). The rate constant for the electrode surface and $^{\bullet}$OH was estimated as the average of the values observed under pH 6 and pH 7 (i.e., 5.7× 10^{5} g^{-1} s^{-1}). The competing

organic compounds were assumed to behave similarly to methanol with respect to $^{\bullet}OH$ scavenging impacts ($k_{\bullet OH}$ = 8.1× 10^4 L mg-C^{-1} s^{-1}).

Text S7. Reaction rate constants for $^{\bullet}OH$ with organic buffer compounds.

Second-order rate constants for the reaction of $^{\bullet}OH$ with MES and PIPES were measured using competition kinetics.[12] Briefly, individual test compounds (MES or PIPES, 1.0 μM) was irradiated in a customized brown glass bottle ($V_{effective}$ = 600 mL), using a 9 W low-pressure Hg UV-C lamp (arc length = 12.5 cm, Anyray, US). The solutions contained 0.5 μM carbamazepine as a reference compound $k_{carbamazepine,\bullet OH}$ = 9.1 × 10^9 M^{-1} s^{-1},[13] along with H_2O_2 as a photosensitizer (10 μM). Carbamazepine was chosen as a reference compound because it exhibits low rate of direct photolysis.[14]

Solution pH was buffered with 100 μM phosphate buffer at 6.0 for the MES experiment and 7.0 for the PIPES experiment, respectively. The pH changed by < 0.2 units through the photolysis experiments. Details of the analytical method are described in Text S8. Because the reaction between the test compounds and H_2O_2 are slow[15] and no H_2O_2 consumption was observed when test compounds were added to H_2O_2-contaning solution (Figure S3), the direct oxidation of the test compounds by H_2O_2 was negligible over the time scale of this study.

Control experiments for direct photolysis of the test compounds and carbamazepine were conducted under similar experimental conditions in the absence of H_2O_2. The pseudo 1st-order rate constants for direct photolysis of carbamazepine and test compounds in the absence of H_2O_2 were less than 10% of the values observed in the presence of H_2O_2 (Figure S18). Due to the low molar absorptivity of MES and PIPES,[16, 17] the contribution of direct photolysis to the overall phototransformation in the presence of H_2O_2 was neglected because the presence of H_2O_2 was expected to further slowdown direct photolysis reactions due to competition for photons.

Assuming that losses of the test compounds and carbamazepine were due only to reaction with •OH, the following equation could be used to estimate the rate of reaction of a buffer compound with •OH ($k_{\mathrm{Buffer},\bullet\mathrm{OH}}$):

$$\ln\left[\frac{[\mathrm{Compound}]_t}{[\mathrm{Compound}]_0}\right] = \frac{k_{\mathrm{Compound},\bullet\mathrm{OH}}}{k_{\mathrm{carbamazepine},\bullet\mathrm{OH}}}\ln\left[\frac{[\mathrm{carbamazepine}]_t}{[\mathrm{carbamazepine}]_0}\right] \qquad \text{(S5)}$$

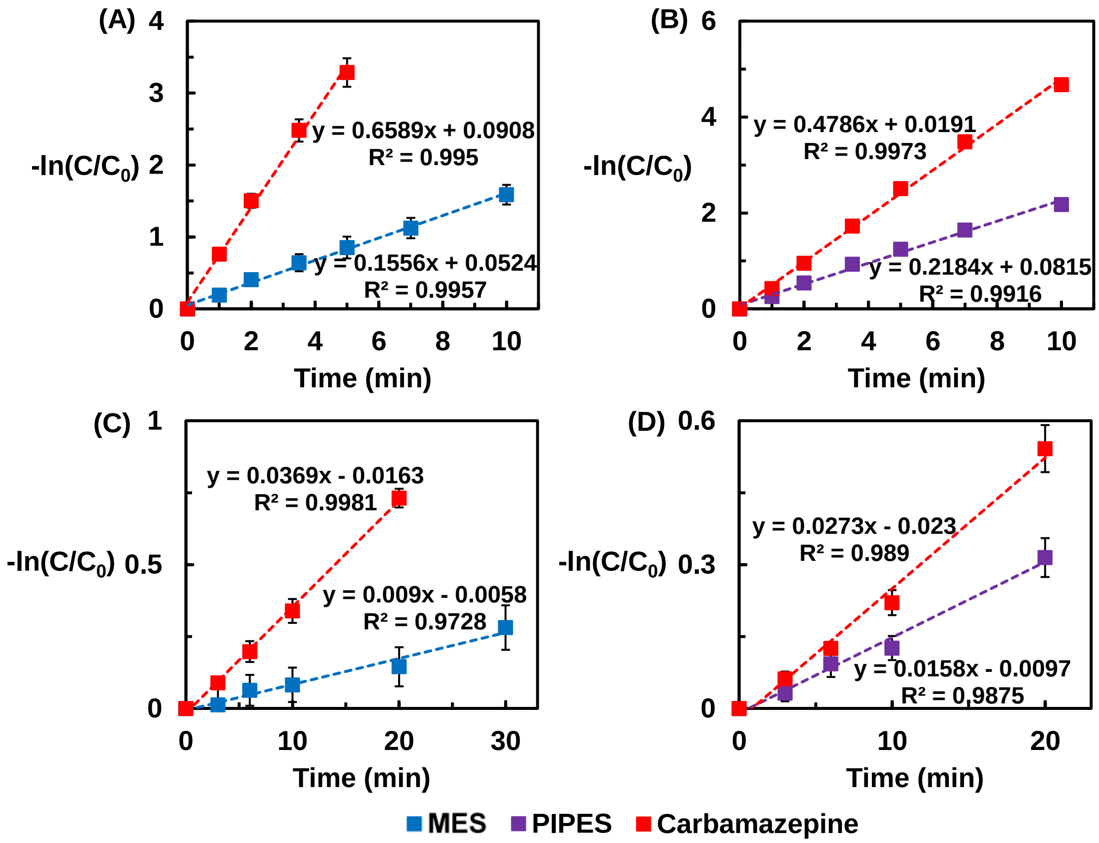

Figure S18. Natural logarithm of normalized concentration versus time for carbamazepine and (A) MES in the presence of H_2O_2 at pH 6, (B) PIPES in the presence of H_2O_2 at pH 7, (C) MES in the absence of H_2O_2 at pH 6 and (D) PIPES in the absence of H_2O_2 at pH 7. [Test compound]$_{\mathrm{init.}}$ = 1.0 μM, [carbamazepine]$_{\mathrm{init.}}$ = 0.5 μM, [H_2O_2]$_{\mathrm{init.}}$ = 10 μM, [PO_4^{3-}] = 100 μM. Error bars represent one standard deviation.

Text S8. Analytical methods for detection of MES and PIPES

100 μL of methanol and 10 μL of isotopically labeled internal standard was added to 1 mL of (diluted) samples right after sample collection to quench any possible •OH reactions that

could consume buffers and carbamazepine. Additionally, 100 µL of 1 N H_2SO_4 was added to all MES-containing samples to obtain a good chromatography.

MES and carbamazepine were separated using a 150 x 3 mm Synergi™ 4 µm Hydro-RP 80 Å column, eluted with at 0.4 mL min^{-1} methanol and 0.1% acetic acid in water with the following gradient: 0 minutes, 0% methanol; 2 minutes, 0% methanol; 8 minutes, 60% methanol; 11 minutes, 95% methanol; 12 minutes, 95% methanol; 12.1 minutes, 0% methanol; 18 minutes, 0% methanol.

Pipes and carbamazepine were separated using a 4.6 mm X 150 mm Waters™ Symmetry C18 100Å, 3.5 µm column, eluted with at 0.4 mL min^{-1} methanol and 0.1% acetic acid in water with the following gradient: 0 minutes, 10% methanol; 3 minutes, 10% methanol; 11 minutes, 95% methanol; 12 minutes, 95% methanol; 15 minutes, 10% methanol; 23 minutes, 10% methanol.

Buffer compounds and carbamazepine were quantified in multiple reaction monitoring (MRM) mode with an Agilent 1260 series HPLC system coupled to a 6460 triple quadrupole tandem mass spectrometer (HPLC-MS/MS) using electrospray ionization (ESI) with a 7-200 ms dwell time and a gas temperature of 350° C, a gas flow rate of 9 L/min at 45 psi, and a capillary voltage of 3600 V. Compound-specific parameters are given in Table S3.

Table S3. Compound-specific mass spectroscopy parameters.

Compound	Precursor ion (amu)	Product ion (amu)	Fragmentor voltage (V)	Collision energy (V)	Ionization mode
Carbamazepine	237	194	120	15	Positive
		179	120	35	
Carbamazepine-d10	247	204	120	20	Positive
MES	196	196	92	0	Positive
PIPES	303	303	92	0	Positive

Text S9. Prediction of reaction rates between H_2O_2 and Fe(II).

The rate of H_2O_2 activation through the reaction with $Fe(II)_{(aq)}$ was calculated by:

$$k_{H_2O_2} = (k_{Fe^{2+}}\alpha_{Fe^{2+}} + k_{FeOH^+}\alpha_{FeOH^+} + k_{Fe(OH)_2^\circ}\alpha_{Fe(OH)_2^\circ})[\mathrm{Fe(II)}] \qquad \text{(S6)}$$

where, $k_{Fe^{2+}} = 76\ M^{-1}\ s^{-1}$, $k_{FeOH^+} = 6.3\times10^6\ s^{-1}\ M^{-1}\ s^{-1}$, and $k_{Fe(OH)_2^\circ} = 7.9\times10^9\ s^{-1}\ M^{-1}\ s^{-1}$,[18-20] and the α_i is the fraction of Fe(II) that exist in the form i and calculated based on:

$$Fe^{2+} + OH^- = FeOH^+ \quad Log\beta_1 = 4.50 \qquad \text{(S7)}$$

$$Fe^{2+} + 2OH^- = Fe(OH)_0^\circ \quad Log\beta_2 = 7.43 \qquad \text{(S8)}$$

Homogeneous Fenton reactions would be expected to activate H_2O_2 at rates constants ranging from 1.4 to 2.0 $\times10^{-3}\ s^{-1}$ at pH values of 4, 5, and 6. Therefore, 35%, 21% and 23% of the H_2O_2 loss could be explained by the homogeneous Fenton pathway at pH 4, 5 and 6, respectively.

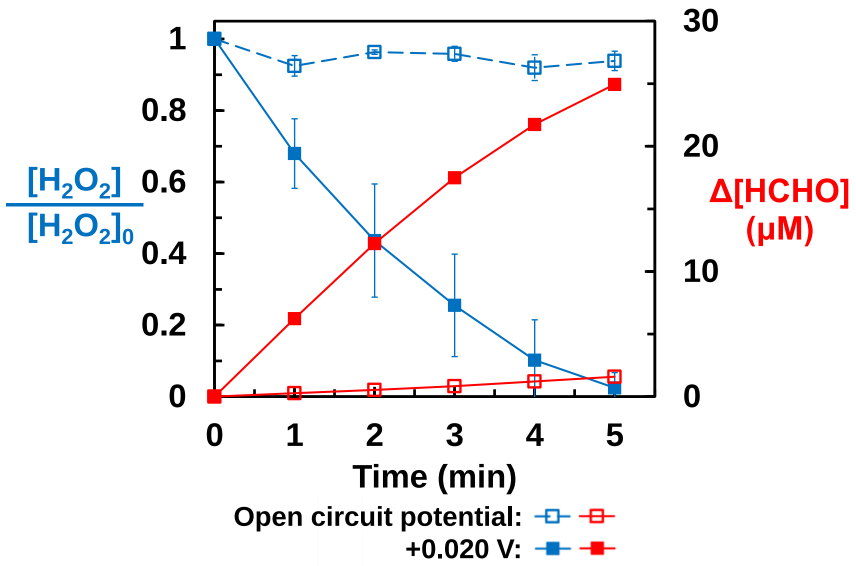

Figure S19. H_2O_2 activation and formaldehyde formation at open circuit potential and +0.020 V. Experiments conducted in buffered Na_2SO_4 electrolyte, pH = 6. $[H_2O_2]_0$ = 1.25 mg/L. Error bars represent one standard deviation.

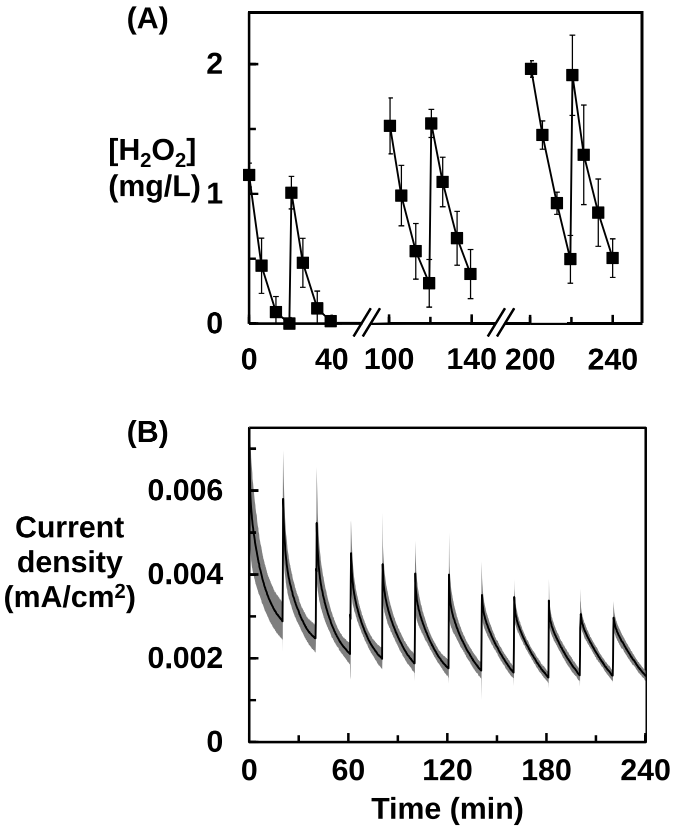

Figure S20. (A) H_2O_2 concentrations and (B) observed current densities during the treatment of an authentic water sample. Potential = +0.020 V, 1.25 mg/L of H_2O_2 was dosed every 20 minutes. Error bars represent one standard deviation.

REFERENCES

1. Duesterberg, C. K.; Cooper, W. J.; Waite, T. D., Fenton-mediated oxidation in the presence and absence of oxygen. *Environmental science & technology* **2005,** *39*, (13), 5052-5058.
2. Keenan, C. R.; Sedlak, D. L., Factors affecting the yield of oxidants from the reaction of nanoparticulate zero-valent iron and oxygen. *Environmental Science & Technology* **2008,** *42*, (4), 1262-1267.
3. Bader, H.; Sturzenegger, V.; Hoigné, J., Photometric method for the determination of low concentrations of hydrogen peroxide by the peroxidase catalyzed oxidation of N,N-diethyl-p-phenylenediamine (DPD). *Water Research* **1988,** *22*, (9), 1109-1115.
4. Viollier, E.; Inglett, P. W.; Hunter, K.; Roychoudhury, A. N.; Van Cappellen, P., The ferrozine method revisited: Fe(II)/Fe(III) determination in natural waters. *Applied Geochemistry* **2000,** *15*, (6), 785-790.
5. US Environmental Protection Agency, Method 7196A: Chromium, hexavalent (colorimetric). In 1992.
6. Barazesh, J. M.; Hennebel, T.; Jasper, J. T.; Sedlak, D. L., Modular Advanced Oxidation Process Enabled by Cathodic Hydrogen Peroxide Production. *Environmental Science & Technology* **2015,** *49*, (12), 7391-7399.
7. Weng, C.; Chuang, Y.-H.; Davey, B.; Mitch, W. A., Reductive Electrochemical Activation of Hydrogen Peroxide as an Advanced Oxidation Process for Treatment of Reverse Osmosis Permeate during Potable Reuse. *Environmental Science & Technology* **2020,** *54*, (19), 12593-12601.
8. Gros, P.; Bergel, A., Improved model of a polypyrrole glucose oxidase modified electrode. *Journal of Electroanalytical Chemistry* **1995,** *386*, (1-2), 65-73.
9. Abdekhodaie, M.; Cheng, J.; Wu, X., Effect of formulation factors on the bioactivity of glucose oxidase encapsulated chitosan–alginate microspheres: In vitro investigation and mathematical model prediction. *Chemical engineering science* **2015,** *125*, 4-12.
10. Chaplin, B. P., The Prospect of Electrochemical Technologies Advancing Worldwide Water Treatment. *Accounts of Chemical Research* **2019,** *52*, (3), 596-604.
11. Chaplin, B. P., Critical review of electrochemical advanced oxidation processes for water treatment applications. *Environmental Science: Processes & Impacts* **2014,** *16*, (6), 1182-1203.
12. Onstein, P.; Stefan, M. I.; Bolton, J. R., Competition Kinetics Method for the Determination of Rate Constants for the Reaction of Hydroxyl Radicals with Organic Pollutants Using the UV/H_2O_2 Advanced Oxidation Technology: The Rate Constants for the tert-Butyl Formate Ester and 2, 4-Dinitrophenol. *Journal of Advanced Oxidation Technologies* **1999,** *4*, 231-236.
13. Jasper, J. T.; Sedlak, D. L., Phototransformation of Wastewater-Derived Trace Organic Contaminants in Open-Water Unit Process Treatment Wetlands. *Environmental Science & Technology* **2013,** *47*, (19), 10781-10790.
14. Pereira, V. J.; Linden, K. G.; Weinberg, H. S., Evaluation of UV irradiation for photolytic and oxidative degradation of pharmaceutical compounds in water. *Water Research* **2007,** *41*, (19), 4413-4423.
15. Zhao, G.; Chasteen, N. D., Oxidation of Good's buffers by hydrogen peroxide. *Analytical Biochemistry* **2006,** *349*, (2), 262-267.

16. Good, N. E.; Winget, G. D.; Winter, W.; Connolly, T. N.; Izawa, S.; Singh, R. M. M., Hydrogen Ion Buffers for Biological Research*. *Biochemistry* **1966,** *5*, (2), 467-477.

17. Taha, M.; Gupta, B. S.; Khoiroh, I.; Lee, M.-J., Interactions of Biological Buffers with Macromolecules: The Ubiquitous “Smart” Polymer PNIPAM and the Biological Buffers MES, MOPS, and MOPSO. *Macromolecules* **2011,** *44*, (21), 8575-8589.

18. Walling, C., Fenton's reagent revisited. *Accounts of chemical research* **1975,** *8*, (4), 125-131.

19. González-Davila, M.; Santana-Casiano, J. M.; Millero, F. J., Oxidation of iron (II) nanomolar with H_2O_2 in seawater. *Geochimica et Cosmochimica Acta* **2005,** *69*, (1), 83-93.

20. King, D. W.; Farlow, R., Role of carbonate speciation on the oxidation of Fe(II) by H_2O_2. *Marine Chemistry* **2000,** *70*, (1), 201-209.
